# Cenozoic climate change and diversification on the continental shelf and slope: evolution of gastropod diversity in the family Solariellidae (Trochoidea)

**DOI:** 10.1002/ece3.513

**Published:** 2013-03-04

**Authors:** S T Williams, L M Smith, D G Herbert, B A Marshall, A Warén, S Kiel, P Dyal, K Linse, C Vilvens, Y Kano

**Affiliations:** 1 Department of Life Sciences, Natural History MuseumCromwell Road, London, SW7 5BD, UK; 2 KwaZulu-Natal MuseumP. Bag 9070, Pietermaritzburg, 3200, South Africa; 3 School of Life Sciences, University of KwaZulu-NatalPietermaritzburg, 3206, South Africa; 4 Museum of New Zealand Te Papa TongarewaPost Office Box 467, Wellington, New Zealand; 5 Swedish Museum of Natural HistoryBox 50007, Stockholm, SE, 10405, Sweden; 6 Geowissenschaftliches Zentrum, Abteilung Geobiologie and Courant Research Center Geobiology, Georg-August Universität GöttingenGoldschmidtstr. 3, 37077, Göttingen, Germany; 7 British Antarctic SurveyHigh Cross, Madingley Road, Cambridge, CB3 0ET, UK; 8 Scientific Collaborator, Muséum national d'Histoire naturelleRue Buffon 55, Paris Cedex 05, 75231, France; 9 Department of Marine Ecosystems Dynamics, Atmosphere and Ocean Research Institute, The University of Tokyo5-1-5 Kashiwanoha, Kashiwa, Chiba, 277-8564, Japan

**Keywords:** Biogeography, deep sea, Eocene–Oligocene transition, phylogeny

## Abstract

Recent expeditions have revealed high levels of biodiversity in the tropical deep-sea, yet little is known about the age or origin of this biodiversity, and large-scale molecular studies are still few in number. In this study, we had access to the largest number of solariellid gastropods ever collected for molecular studies, including many rare and unusual taxa. We used a Bayesian chronogram of these deep-sea gastropods (1) to test the hypothesis that deep-water communities arose onshore, (2) to determine whether Antarctica acted as a source of diversity for deep-water communities elsewhere and (3) to determine how factors like global climate change have affected evolution on the continental slope. We show that although fossil data suggest that solariellid gastropods likely arose in a shallow, tropical environment, interpretation of the molecular data is equivocal with respect to the origin of the group. On the other hand, the molecular data clearly show that Antarctic species sampled represent a recent invasion, rather than a relictual ancestral lineage. We also show that an abrupt period of global warming during the Palaeocene Eocene Thermal Maximum (PETM) leaves no molecular record of change in diversification rate in solariellids and that the group radiated before the PETM. Conversely, there is a substantial, although not significant increase in the rate of diversification of a major clade approximately 33.7 Mya, coinciding with a period of global cooling at the Eocene–Oligocene transition. Increased nutrients made available by contemporaneous changes to erosion, ocean circulation, tectonic events and upwelling may explain increased diversification, suggesting that food availability may have been a factor limiting exploitation of deep-sea habitats. Tectonic events that shaped diversification in reef-associated taxa and deep-water squat lobsters in central Indo-West Pacific were also probably important in the evolution of solariellids during the Oligo-Miocene.

## Introduction

The deep sea is the largest and most enigmatic of the Earth's ecosystems (Ramirez-Llodra et al. 2010). Covering almost two-thirds of the planet's surface, it was once thought to be devoid of life. Studies over the last hundred years, however, have shown that the deep sea is in fact rich in species, some with bizarre and novel adaptations to the challenges of living at great depth. This understudied but important marine environment is at risk from overexploitation and habitat destruction as a result of both fishing and mining ventures (e.g., Halfar and Fujita 2007; Van Dover 2011), and it is vital that we learn more about the diversity of its biota and their evolution before these habitats suffer further destruction.

Elucidating the factors driving diversification in the deep sea is of profound importance if we are to understand how deep-sea groups have evolved. Climate change has been shown to result in shifts in primary producers that also affect deep-sea community structure over periods of a few years (Ruhl and Smith 2004), over hundreds of thousands of years spanning the past four glacial–interglacial cycles (Yasuhara et al. 2009, 2012) and over millions of years (Smith and Stockley 2005). It is likely therefore that climate change over geological time has also played an important role in the evolution of deep-sea diversity (e.g., Lipps and Mitchell 1976; Gingerich 2006; Berger 2007).

The Palaeocene/Eocene boundary (∼55.8 Mya) was marked by a brief but intense global warming event, known as the Palaeocene-Eocene Thermal Maximum (PETM), which saw global temperatures rise by 5°C over 10,000 years. Sea surface temperatures rose between 5°C in the tropics and 9°C in the high latitudes, and bottom-water temperatures by 4–5°C over a period of about 10,000 years (Zachos et al. 2001, 2008). The event was also associated with a massive injection of ^13^C-depleted carbon into the ocean-atmosphere system (Dickens et al. 1995), resulting in shallowing of the calcite compensation depth (CCD) and acidification in the deep sea (Zachos et al. 2005). This in turn is thought to have contributed to the contemporaneous mass extinction of benthic Foraminifera, although most plankton survived (Tjalsma and Lohmann 1983; Zachos et al. 2005) and a transient diversification was observed in topical, planktonic foraminifera (Kelly et al. 1998). On land, this dramatic climate change resulted in a rapid increase in plant speciation and diversity in tropical habitats (Jaramillo et al. 2010), a turnover in large mammals in northern continents and possibly Africa (Gingerich 2006; Blois and Hadley 2009) and a rapid and transient northward migration of plants in North America (Wing et al. 2005). The PETM was followed by the Eocene Optimum, an extended period of very warm temperatures (Zachos et al. 2001, 2008).

Another dramatic change in global climate occurred at the Eocene–Oligocene transition (EOT), when there was a period of abrupt cooling lasting about 500 kyr between 33.5 and 34 Mya (Zachos et al. 2001, 2008; Pearson et al. 2008). Atmospheric and high latitude sea-surface temperatures cooled by ∼5°C (Kennett and Shackleton 1976; Liu et al. 2009) and both the thermocline and deep water of the Southern Ocean and Indian Ocean cooled by 2–3°C across the EOT (Dunkley Jones et al. 2008; Bohaty et al. 2012). The Earth shifted from “greenhouse” to “icehouse” conditions with large, permanent ice-sheets forming in Antarctica (Zachos et al. 1996; Lear et al. 2000). The growth of a continental-scale ice sheet in Antarctic is thought to have been a primary driver of changes to Antarctic circulation, which in turn caused increased latitudinal thermal gradients, increased thermohaline circulation, increased deep-basin ventilation, decreased deep-ocean acidity, a deepening of the CCD and intensified upwelling that coincided with periods of sea-level fluctuations (van Andel 1975; Coxall et al. 2005; Rea and Lyle 2005; Berger 2007; Miller et al. 2009). The onset of the Antarctic Circumpolar Current (ACC) also occurred concurrently with the EOT and tectonic events leading to the opening of both the Drake and Tasman Passages (Katz et al. 2011).

Intense chemical weathering of siliceous rocks at high latitudes is thought to have occurred prior to the EOT during the warm climate of the Eocene Optimum, releasing high concentrations of silica into the oceans (Lear et al. 2000). The ACC triggered mixing of deep-water layers around Antarctica, leading to an increase in silica and other nutrients in the deep sea (Lear et al. 2000; Berger 2007; Marx and Uhen 2010). The increase in nutrients is thought to have resulted in diversification of siliceous diatoms (Miller et al. 2009), which in turn is thought to have resulted in increased abundance of krill, leading to the diversification of whales in southern oceans (Berger 2007; Marx and Uhen 2010).

Little is known about the origin of deep-sea clades, but fossil evidence suggests that post-Palaeozoic order-level benthic marine taxa first appeared near or onshore, even if they are now found only in the deep sea (Jablonski et al. 1983; Jablonski and Bottjer 1991; Jablonski 2005). Iconic examples for this include primitive taxa such as monoplacophorans and stalked crinoids that dominated shallow marine environments during the Palaeozoic, but currently occur only in deep-sea refugia (Lemche 1957; Ameziane and Roux 1997; Aronson and Blake 2001; Kano et al. 2012). Conversely, the origins of lower level taxa such as families and genera do not always conform to this pattern (Jablonski 2005). Indeed, molecular evidence suggests radiations move from offshore to onshore (e.g., deep-sea corals, Lindner et al. 2008) as well as in the reverse direction (e.g., isopods, Raupach et al. 2012). Previous authors have suggested the shift in origin may have been due to deep-sea anoxic events that were frequent prior to the Cenozoic (Jacobs and Lindberg 1998); however, this is now debated (Jablonski 2005). An additional hypothesis is that early deep-sea radiations originated in shallow-water, but were displaced into deep-water as a result of pressure from predators or competitors (Vermeij 1987).

Another theory is that since the onset of glacial climates, Antarctica may have acted as a center of origin for deep-sea taxa, with Antarctic shelf taxa moving into deep-water as a result of climatic deterioration during glaciation periods and the subsequent loss of shallow-water habitat (Zinsmeister and Feldmann 1984; Crame 1993; Rogers 2000; Briggs 2003; Brandt et al. 2007; Strugnell et al. 2008). Range expansion of Antarctic marine organisms into the Southern Ocean followed the development of the Antarctic Circumpolar Current (ACC; ∼33.8 Mya) and the northward movement of Antarctic bottom water (20–5 Mya; Lawver and Gahagan 2003). The ACC connected shallow-water Antarctic fauna with deep-water in the Atlantic, Indian and Pacific Oceans contributing to the Cenozoic diversification in the Southern Ocean (Brey et al. 1996; Rogers 2000; Briggs 2003; Brandt et al. 2007; Strugnell et al. 2008; Clarke and Crame 2010).

We used a deep-sea radiation of vetigastropods as a tractable model to test these key hypotheses about origins and to determine the factors driving diversification on the continental slope. The marine gastropod family Solariellidae is a group of small (5 mm–2 cm) marine snails that occur globally, predominantly in deep-water, although some species occur as shallow as 5 m (Warén 1993). Specifically, our objectives were to use Bayesian inference to estimate species trees and divergence times, with palaeontological data informing calibration of key nodes in the tree. The resulting chronogram was used: (1) to test the hypothesis that deep-water communities arose from shallow-water ancestors; (2) to determine whether Antarctic shallow-water species represent new invasions or relictual ancestors of lineages that acted as a source of diversity for deep-water communities elsewhere; and (3) to examine the timing of diversification in order to determine the factors driving evolution in the deep sea. Two factors were of special interest: the two shifts in global temperature discussed above and tectonic activity in Southeast Asia. The latter has been shown to drive diversification in both shallow and deep-water groups in the Indo-West Pacific (Kohn 1990; Wilson and Rosen 1998; Williams 2007; Renema et al. 2008; Williams and Duda 2008; Bellwood et al. 2012; Cabezas et al. 2012) and we would expect to see a similar pattern in solariellids. We would predict that the PETM would have had little effect on deep-sea organisms over the depth distribution of solariellids, as they may have been less affected by dramatic temperature increases than intertidal and terrestrial organisms and most solariellid species occur above the CCD. Conversely, we would predict that events contemporaneous with the EOT might have led to increased diversification in Southern Ocean and Indo-West Pacific (IWP) solariellids, reflecting diversification patterns of other marine taxa in the Southern Ocean (e.g., Berger 2007; Miller et al. 2009; Marx and Uhen 2010).

## Materials and Methods

### Samples

Recently, MNHN deep-sea expeditions have obtained unprecedented collections of solariellids from New Caledonia, Vanuatu, Solomon Islands, Philippines, Norfolk Ridge, Chesterfield Bank, Papua New Guinea, Madagascar and Mozambique Channel, all of which were included in this study. Additional specimens from Japan, Antarctica, Norway, New Zealand, South Africa, and Australia were collected by the authors or loaned from other museums. Sequences were obtained from a total of 208 solariellid specimens and 25 outgroup taxa (Table 1 for solariellids, Table S1 for outgroup taxa). Based on recent revisions, our study has included all but two genera: the IWP genera *Minolops* (which may be synonymous with *Spectamen*; Marshall 1999) and one Atlantic genus, *Microgaza*. The choice of outgroup taxa was based on Williams (2012).

**Table 1:** Solariellid specimens used in study, ordered by genera or clades identified in this study, along with details of sampling localities (expedition name, station number, detailed sampling locality, depth range of trawl or dredge and longitude and latitude of start point of dredge), registration numbers of voucher specimens, and EMBL accession numbers for sequences

Species	Expedition/Station	Sample locality	Depth	Lat/Long	Reg	28S	COI	16S	12S
*Archiminolia* 1	SALOMON2/DW2301	S Gatukai I., Solomon Islands	267–329 m	9°6.9′S, 158°20.6′E	MNHN 200718540	HF586167	HF586310	HF586019	HF585858
*Archiminolia* 2	EBISCO/CP2572	N Bellona, New Caledonia	324–330 m	20°23′S, 158°45′E	MNHN 200718316	HF586168	HF586311	HF586020	HF585859
*Archiminolia* 2	EBISCO/DW2522	S Banc Nova, New Caledonia	310–318 m	22°46′S, 159°21′E	MNHN 200718321	HF586169	HF586312	HF586021	HF585860
*Archiminolia* 2	EBISCO/DW2532	N Banc Nova, New Caledonia	350 m	22°15′S, 159°27′E	MNHN 200734079	HF586174	HF586317	HF586026	HF585866
*Archiminolia* 2	NORFOLK2/DW2091	Banc Antigonia, Norfolk Ridge	600–896 m	24°45′S, 168°06′E	MNHN 200718338	HF586170	HF586313	HF586022	HF585861
*Archiminolia* 2	NORFOLK2/DW2117	Banc Kaimon Maru, Norfolk Ridge	400 m	23°24′S, 168°00′E	MNHN 200718339	–	–	–	HF585862
*Archiminolia* 2	RV Tangaroa/2003020	Norfolk Ridge, S of Norfolk I., Australia	322–337 m	29°41.8′S, 168°2.6′E	NMNZ M171105	–	–	–	HF585867
*Archiminolia* 2	TERRASSES/DW3108	Munida, Norfolk Ridge	370–440 m	23°01′S, 168°23′E	MNHN 20098803	HF586171	HF586314	HF586023	HF585863
*Archiminolia* 2	TERRASSES/DW3063	Banc Antigonia, Norfolk Ridge	430–480 m	23°23′S, 168°00′E	MNHN 20098804	HF586172	HF586315	HF586024	HF585864
*Archiminolia* 2	TERRASSES/DW3107	Munida, Norfolk Ridge	380–440 m	23°01′S, 168°23′E	MNHN 20098867	HF586173	HF586316	HF586025	HF585865
*Bathymophila* 1	EBISCO/CP2571	N Bellona, New Caledonia	298–309 m	20°25′S, 158°45′E	MNHN 200718313	HF586073	HF586214	HF585923	HF585747
*Bathymophila* 1	EBISCO/DW2639	N Lansdowne, New Caledonia	289–294 m	20°47′S, 161°01′E	MNHN 200734144	HF586074	HF586215	HF585924	HF585748
*Bathymophila* 1	EBISCO/DW2639	S Lansdowne, New Caledonia	289–294 m	20°47′S, 161°01′E	MNHN 200734145	HF586075	HF586216	HF585925	HF585749
*Bathymophila* 2	CONCALIS/DW2993	Grand Passage, New Caledonia	700–730 m	18°00′S, 163°02′E	MNHN 200735577	HF586077	HF586218	HF585927	HF585751
*Bathymophila* 2	CONCALIS/DW3023	Grand Passage, New Caledonia	285–300 m	19°00′S, 163°26′E	MNHN 200735589	HF586078	–	HF585928	HF585752
*Bathymophila* 2	EBISCO/DW2584	Chesterfield, New Caledonia	569–570 m	19°38′S, 158°44′E	MNHN 200718323	HE800722	HE800623	HE800762	HE800673
*Bathymophila diadema*	BIOPAPUA/CP3755	Off Bougainville, Papua New Guinea	662 m	5°04′S, 154°29′E	MNHN 200915191	HF586088	HF586229	HF585938	HF585764
*Bathymophila diadema*	CONCALIS/DW2983	Grand Passage, New Caledonia	367–430 m	18°01′S, 163°02′E	MNHN 200735575	HF586087	HF586228	HF585937	HF585763
*Bathymophila diadema*	EBISCO/CP2556	W Bellona, New Caledonia	741–791 m	21°06′S, 158°32′E	MNHN 200718311	HF586079	HF586219	HF585929	HF585753
*Bathymophila diadema*	EBISCO/CP2556	W Bellona, New Caledonia	741–791 m	21°06′S, 158°32′E	MNHN 200718312	HF586080	HF586220	HF585930	HF585754
*Bathymophila diadema*	EBISCO/CP2651	SE Fairway, New Caledonia	883–957 m	21°29′S, 162°36′E	MNHN 200718319	HF586081	HF586221	HF585931	HF585755
*Bathymophila diadema*	EBISCO/CP2651	SE Fairway, New Caledonia	883–957 m	21°29′S, 162°36′E	MNHN 200718320	HF586082	HF586222	HF585932	HF585756
*Bathymophila diadema*	EBISCO/DW2544	W Bellona, New Caledonia	650–723 m	21°10′S, 158°39′E	MNHN 200718322	HE800721	HE800622	HE800761	HE800672
*Bathymophila diadema*	SALOMON2/CP2249	NW Vella, Lavella I., Solomon Islands	782–884 m	7°31.3′S, 156°17.7′E	MNHN 200718535	–	HF586223	–	HF585725
*Bathymophila diadema*	SALOMON2/CP2249	NW Vella, Lavella I., Solomon Islands	782–884 m	7°31.3′S, 156°17.7′E	MNHN 200913010	–	–	–	HF585757
*Bathymophila diadema*	SALOMON2/CP2249	NW Vella, Lavella I., Solomon Islands	782–884 m	7°31.3′S, 156°17.7′E	MNHN 200913011	–	HF586224	–	HF585758
*Bathymophila diadema*	TERRASSES/DW3040	Mont J, Loyalty Ridge	750–780 m	23°58′S, 169°43′E	MNHN 20098802	HF586083	HF586225	HF585933	HF585760
*Bathymophila diadema*	TERRASSES/DW3045	Mont J, Loyalty Ridge	660–710 m	23°48′S, 169°46′E	MNHN 20098869	HF586084	HF586226	HF585934	HF585761
*Bathymophila diadema*	TERRASSES/DW3045	Mont J, Loyalty Ridge	660–710 m	23°48′S, 169°46′E	MNHN 20098871	HF586085	HF586227	HF585935	HF585762
*Bathymophila diadema*	CONCALIS/DW2983	Grand Passage, New Caledonia	367–430 m	18°01′S, 163°02′E	MNHN 200735574	HF586086	–	HF585936	–
*Bathymophila* 4	MIRIKY/CP3221	Between Nosy-bé and Banc du Leven, Madagascar	782 m	12°47′S, 48°08′E	MNHN 20098762	HF586089	HF586230	HF585939	HF585765
*Bathymophila* 4	MIRIKY/CP3221	Between Nosy-bé and Banc du Leven, Madagascar	782 m	12°47′S, 48°08′E	MNHN 20098763	HF586090	HF586231	HF585940	HF585766
*Bathymophila* 4	MIRIKY/CP3221	Between Nosy-bé and Banc du Leven, Madagascar	782 m	12°47′S, 48°08′E	MNHN 20098764	HF586091	HF586232	–	HF585767
*Bathymophila* 4	MIRIKY/CP3192	Between Nosy-bé and Banc du Leven, Madagascar	578–580 m	12°26′S, 48°13′E	MNHN 20098769	HF586092	HF586233	HF585941	HF585768
*Bathymophila* 4	MIRIKY/CP3186	Between Nosy-bé and Banc du Leven, Madagascar	613–625 m	12°34′S, 48°09′E	MNHN 20098770	HF586093	HF586234	HF585942	HF585769
*Bathymophila* 4	MIRIKY/CP3192	Between Nosy-bé and Banc du Leven, Madagascar	578–580 m	12°26′S, 48°13′E	MNHN 20098771	HF586094	HF586235	HF585943	HF585770
*Bathymophila* 4	MIRIKY/CP3221	Between Nosy-bé and Banc du Leven, Madagascar	782 m	12°47′S, 48°08′E	MNHN 20098772	HF586095	HF586236	HF585944	HF585771
*Bathymophila* 4	MIRIKY/CP3221	Between Nosy-bé and Banc du Leven, Madagascar	782 m	12°47′S, 48°08′E	MNHN 20098773	HF586096	HF586237	HF585945	HF585772
*Bathymophila* 5	AURORA/CP2683	Philippines	1743–1754 m	15°06′N, 123°04′E	MNHN 200718295	HF586076	HF586217	HF585926	HF585750
*Bathymophila* 6	CONCALIS/DW2990	Grand Passage, New Caledonia	650–700 m	17°59′S, 163°03′E	MNHN 200735547	HF586097	HF586238	HF585946	HF585773
*Bathymophila* 7	EBISCO/CP2651	SE Fairway, New Caledonia	883–957 m	21°29′S, 162°36′E	MNHN 200718317	HF586098	HF586239	HF585947	HF585774
*Bathymophila cf callomphala*	CONCALIS/DW2980	Grand Passage, New Caledonia	574–660 m	18°16′S, 162°57′E	MNHN 200735553	HF586099	HF586240	HF585948	HF585778
*Bathymophila* 9	CONCALIS/DW3023	Grand Passage, New Caledonia	285–300 m	19°00′S, 163°26′E	MNHN 200735590	HF586100	HF586241	HF585949	HF585779
*Bathymophila* 10	BIOPAPUA/CP3724	Vitiaz Straight, Papua New Guinea	860–880 m	05°59′S, 147°39′E	MNHN 200915182	HF586101	HF586242	HF585950	HF585781
*Bathymophila* 11	TARASOC/DW3369	Niau, Tuamotu Archipelago	412–520 m	16°08′S, 146°24′W	MNHN 200915175	HF586102	HF586243	HF585951	HF585787
*Bathymophila* 12	BORDAU1/DW1469	Fiji	314–377 m	19°40′S, 178°10′W	MNHN 200928741	–	–	–	HF585775
*Bathymophila alabida*	RV Karehoa/2000044	S Kermadec Ridge, Rumble III volcano, New Zealand	523 m	35°43.4′S, 178°29.3′E	NMNZ M299686	–	–	–	HF585776
*Bathymophila* 14	BORDAU1/DW1432	Fiji	477–493 m	17°20′S, 178°44′W	MNHN NR	–	–	–	HF585777
*Bathymophila* 15	T/V Nagasaki-maru, N226/Dredge A	SW of Nagasaki, Kyushu I., Japan	470–487 m	32º 10′ N, 129º 30′ E	YK1383	HF586103	HF586244	HF585952	HF585782
*Bathymophila* 16	BOA1/CP2473	Between Ambrim and Malekula, Vanuatu	657–685 m	16º 19′ S, 167º 47′ E	YK1385	HF586104	–	HF585953	HF585783
*Bathymophila* 17	BENTHAUS/DW1951	Lotus Bank, Austral Is.	206–450 m	23°49′S, 147°53′W	MNHM 20095062	–	–	GQ160692	–
Clade A sp 1	BIOPAPUA/DW3688	Seamount S of Manus I., Papua New Guinea	402–640 m	3°04′S, 147°32′E	MNHN 200915186	HF586157	HF586300	–	HF585846
Clade A sp 1	BIOPAPUA/DW3687	Seamount S of Manus I., Papua New Guinea	305–579 m	3°04′S, 147°32′E	MNHN 200915188	HF586158	HF586301	HF586010	HF585847
Clade A sp 1	BIOPAPUA/DW3687	Seamount S of Manus I., Papua New Guinea	305–579 m	3°04′S, 147°32′E	MNHN 200915189	HF586159	HF586302	HF586011	HF585848
Clade A *tenorioi*	PANGLAO2005/CP2394	Off Balicasag I., Philippines	470–566 m	9°28.6′N, 123°40′E	MNHN 200718423	–	HF586305	HF586015	HF585853
Clade A *tenorioi*	PANGLAO2005/CP2394	Off Balicasag I., Philippines	470–566 m	9°28.6′N, 123°40′E	MNHN 200718424	HF586163	HF586306	HF586016	HF585854
Clade A *tenorioi*	PANGLAO2005/CP2394	Off Balicasag I., Philippines	470–566 m	9°28.6′N, 123°40′E	MNHN 200718425	HF586164	HF586307	–	HF585855
Clade A *tenorioi*	PANGLAO2005/CP2394	Off Balicasag I., Philippines	470–566 m	9°28.6′N, 123°40′E	MNHN 200718429	HF586165	HF586308	HF586017	HF585856
Clade A *tenorioi*	PANGLAO2005/CP2399	Bohol Sea, off Balicasag I., Philippines	309–342 m	9°31.7′N, 123°41.9′E	MNHN 200718394	HF586166	HF586309	HF586018	HF585843
Clade A sp 3	BIOPAPUA/CP3721	Vitiaz Straight, Papua New Guinea	542–554 m	6°03′S, 147°37′E	MNHN 200915183	HF586156	HF586299	HF586008	HF585780
Clade A sp 4	NORFOLK2/DW2057	Norfolk Ridge	555–565 m	24°40′S, 168°39′E	MNHN 200917849	HF586160	–	HF586013	–
Clade A sp 4	NORFOLK2/DW2057	Norfolk Ridge	555–565 m	24°40′S, 168°39′E	MNHN 200917850	HF586161	HF586304	HF586014	HF585850
Clade A sp 4	NORFOLK2/DW2057	Norfolk Ridge	555–565 m	24°40′S, 168°39′E	MNHN 200917851	–	–	–	HF585851
Clade A sp 4	NORFOLK2/DW2057	Norfolk Ridge	555–565 m	24°40′S, 168°39′E	MNHN 200917852	HF586162	–	–	HF585852
Clade A sp 5	AURORA/CP2695	Philippines	357–367 m	14°46′N, 123°40′E	MNHN 200718282	–	HF586303	HF586012	HF585849
Clade A sp 6	CSIRO RV “Southern Surveyor”/SS1005/012	Perth Canyon, Western Australia	479–484 m	31.92°S, 115.02°E	WAM S25773	–	HF586298	–	HF585842
Clade A sp 7	NORFOLK1/DW1679	Kaimon Maru Bank, Norfolk Ridge	298–324 m	24°43′S, 168°10′E	MNHN 200928739	–	–	–	HF585844
Clade A sp 7	NORFOLK1/DW1691	Eponge Bank, Norfolk Ridge	509–513 m	24°54′S, 168°22′E	MNHN 200928740	–	–	HF586009	HF585845
Clade B *iridescens*	–	Off Shionomisaki, Wakayama Pref., Japan	300 m	33°24.8′N, 135°42′E	No voucher	EU530041	–	–	–
Clade B sp 2	MAINBAZA/CP3140	Maputo transect, Mozambique Channel	886–898 m	23°33′S, 36°02′E	MNHN 20098739	HE800720	HE800621	HE800760	HE800671
Clade B sp 2	MAINBAZA/CP3140	Maputo transect, Mozambique Channel	886–898 m	23°33′S, 36°02′E	MNHN 20098742	HF586070	HF586211	HF585920	HF585744
Clade B sp 2	MAINBAZA/CP3140	Maputo transect, Mozambique Channel	886–898 m	23°33′S, 36°02′E	MNHN 20098744	HF586071	HF586212	HF585921	HF585745
Clade B sp 3	T/V Nagasaki-maru, N295/A-1	SW of Nagasaki, Kyushu I., Japan	498–503 m	32º 09′ N, 129º 31′ E	YK1407	HF586072	HF586213	HF585922	HF585746
Clade C sp. 1	SALOMON1/CP1804	Solomon Islands	309–328 m	9°32.0′S, 160°37.4′E	MNHN 200718507	HF586057	–	HF585906	HF585857
Clade C sp. 1	SALOMON1/CP1804	Solomon Islands	309–328 m	9°32.0′S, 160°37.4′E	MNHN 200913304	–	–	–	HF585759
Clade C sp. 1	SALOMON1/CP1804	Solomon Islands	309–328 m	9°32.0′S, 160°37.4′E	MNHN 200943074	–	–	–	HF585724
Clade C sp. 1	SALOMON1	Solomon Islands	–	–	MNHN NR	–	–	–	HF585727
Clade C sp. 2	BOA1/CP2466	SE Malekula, Vanuatu	786–800 m	16°44′S, 167°59′E	MNHN 200718302	–	–	HF585907	HF585728
Clade C sp. 2	EBISCO/CP2651	SE Fairway, New Caledonia	883–957 m	21°29′S, 162°36′E	MNHN 200718318	HF586058	–	HF585908	HF585729
Clade C sp. 2	SALOMON2/CP2249	NW Vella, Lavella I., Solomon Islands	782–884 m	7°31′S, 156°18′E	MNHN 200913008	HF586059	–	HF585909	HF585730
Clade C sp. 2	SALOMON2/CP2249	NW Vella, Lavella I., Solomon Islands	782–884 m	7°31′S, 156°18′E	MNHN 200913009	–	–	–	HF585731
Clade C sp. 3	PANGLAO2005/CP2398	Bohol Sea, off Balicasag I., Philippines	713–731 m	9°32.6′N, 123°40.5′E	MNHN 200718426	HF586060	HF586206	HF585910	HF585732
Clade C sp. 4	SALOMON2/DW2259	Kolombangara I., Vella Gulf, Solomon Islands	396–423 m	8°03.7′S, 156°55.0′E	MNHN 200718539	HF586061	HF586207	HF585911	HF585733
Clade C sp. 4	SALOMON2/DW2259	Kolombangara I., Vella Gulf, Solomon Islands	396–423 m	8°03.7′S, 156°55.0′E	MNHN 200943075	–	–	–	HF585734
Clade C sp. 5	BIOPAPUA/DW3749	Seamount off Bougainville, Papua New Guinea	620–663 m	5°39′S, 153°59′E	MNHN 200915184	HF586064	–	HF585914	HF585738
Clade C sp. 5	BIOPAPUA/DW3749	Seamount off Bougainville, Papua New Guinea	620–663 m	5°39′S, 153°59′E	MNHN 200915185	HF586065	–	HF585915	HF585739
Clade C sp. 5	BIOPAPUA/CP3760	Off Feni Is, Papua New Guinea	613–660 m	3°58′S, 153°43′E	MNHN 200915192	HF586066	HF586209	HF585916	HF585740
Clade C sp. 5	BIOPAPUA/CP3740	Off Woodlark I., Papua New Guinea	556–645 m	9°12′S, 152°16′E	MNHN 200915193	HF586067	–	HF585917	HF585741
Clade C sp. 5	SALOMON1/DW1772	Solomon Islands	570–756 m	8°15.8′S, 160°40.4′E	MNHN 200718508	HF586062	–	HF585912	HF585735
Clade C sp. 5	SALOMON2/CP2243	W Vella, Lavella I., Solomon Islands	518–527 m	7°42.9′S, 156°27.3′E	MNHN 200718534	–	–	–	HF585737
Clade C sp. 5	SALOMON2/CP2243	W Vella, Lavella I., Solomon Islands	518–527 m	7°42.9′S, 156°27.3′E	MNHN 200943073	HF586063	HF586208	HF585913	HF585736
Clade C sp. 6	BIOPAPUA/CP3759	Papua New Guinea	287–352 m	4°00′S, 153°36′E	MNHN 200915195	HF586068	–	HF585918	HF585742
Clade C sp. 7	T/V Nagasaki-maru, N226/Dredge A	SW of Nagasaki, Kyushu I., Japan	470–487 m	32º 10′ N, 129º 30′ E	YK1384	HF586069	HF586210	HF585919	HF585743
Clade C sp. 8	EBISCO/CP2623	Lansdowne, New Caledonia	691–886 m	20°06′S, 160°19′E	MNHN 200943077	–	–	–	HF585722
Clade C sp. 8	TERRASSES/DW3041	Loyalty Ridge, Mont J, New Caledonia	800–840 m	23°59′S, 169°44′E	MNHN 20098874	HF586055	–	HF585904	HF585721
Clade C sp. 8	TERRASSES/DW3036	Loyalty Ridge, Walpole, New Caledonia	800 m	22°41′S, 168°58′E	MNHN 20098876	HF586056	HF586205	HF585905	HF585723
Clade C sp. 8	TERRASSES/DW3045	Loyalty Ridge, Mont J, New Caledonia	660–710 m	23°48′S, 169°46′E	MNHN 20098861	HE800719	HE800620	HE800759	HE800670
*Ilanga* 1	BIOPAPUA/DW3745	Seamount off Bougainville, Papua New Guinea	369–377 m	5°33′S, 154°00′E	MNHN 200915190	HF586107	HF586246	HF585957	HF585789
*Ilanga* 1	BIOPAPUA/DW3745	Seamount off Bougainville, Papua New Guinea	369–377 m	5°33′S, 154°00′E	MNHN 200915197	HF586108	HF586247	HF585958	HF585790
*Ilanga discus*	MIRIKY/CP3188	Between Nosy-bé and Banc du Leven, Madagascar	298–301 m	12°31′S, 48°22′E	MNHN 20098758	HF586109	HF586248	HF585959	HF585791
*Ilanga discus*	MIRIKY/CP3188	Between Nosy-bé and Banc du Leven, Madagascar	298–301 m	12°31′S, 48°22′E	MNHN 20098760	HF586110	HF586249	HF585960	HF585792
*Ilanga discus*	MIRIKY/CP3188	Between Nosy-bé and Banc du Leven, Madagascar	298–301 m	12°31′S, 48°22′E	MNHN 20098761	HE800724	HE800625	HE800764	HE800675
*Ilanga discus*	MIRIKY/CP3188	Between Nosy-bé and Banc du Leven, Madagascar	298–301 m	12°31′S, 48°22′E	MNHN 20098776	HF586111	HF586250	HF585961	HF585793
*Ilanga discus*	MIRIKY/CP3188	Between Nosy-bé and Banc du Leven, Madagascar	298–301 m	12°31′S, 48°22′E	MNHN 20098777	HF586112	HF586251	HF585962	HF585794
*Ilanga* 3	BOA1/CP2413	Malo I., Vanuatu	268–445 m	15°42′S, 167°02′E	MNHN 200718301	HF586114	HF586253	HF585964	HF585796
*Ilanga* 4	T/V Nagasaki-maru, N295/AA	E of Fukue I., Goto Is, Kyushu, Japan	235–238 m	32º 30′ N, 129º 08′ E	YK1380	HF586150	HF586291	HF586000	HF585837
*Ilanga* 4	R/V Tansei-maru, KT-11-12/T5	Off Cape Toi, Miyazaki, Kyushu I., Japan	207–216 m	31º 09′ N, 131º 26′ E	YK1485	–	HF586292	–	–
*Ilanga* 4	TAIWAN2001/CP76	Off Tashi, NE Coast of Taiwan	115–170 m	24°57′N, 122°02′E	MNHN 200718548	–	–	–	HF585803
*Ilanga* 5	CONCALIS/CP2961	Grand Passage, New Caledonia	220–390 m	19°04′S, 163°11′E	MNHN 200735552	HF586122	HF586263	HF585973	HF585806
*Ilanga* 5	CONCALIS/CP2961	Grand Passage, New Caledonia	220–390 m	19°04′S, 163°11′E	MNHN 200735578	HF586123	HF586264	HF585974	HF585807
*Ilanga* 5	CONCALIS/CP2961	Grand Passage, New Caledonia	220–390 m	19°04′S, 163°11′E	MNHN 200735579	HF586124	HF586265	HF585975	HF585808
*Ilanga* 5	CONCALIS/CP2961	Grand Passage, New Caledonia	220–390 m	19°04′S, 163°11′E	MNHN 200735584	HF586125	HF586266	HF585976	HF585809
*Ilanga* 6	SANTO2006/AT112	W Tutuba I., Vanuatu	150–168 m	15°33.5′S, 167°16.1′E	MNHN 200718446	HF586127	HF586268	HF585978	HF585812
*Ilanga* 6	SANTO2006/–	Vanuatu	No data	No data	MNHN 200718447	HF586128	HF586269	HF585979	HF585813
*Ilanga cf. norfolkensis*	CONCALIS/CP3010	Grand Passage, New Caledonia	603 m	18°46′S, 163°19′E	MNHN 200735580	HF586131	HF586272	HF585982	HF585816
*Ilanga cf. norfolkensis*	CONCALIS/CP3010	Grand Passage, New Caledonia	603 m	18°46′S, 163°19′E	MNHN 200735581	HF586132	HF586273	HF585983	HF585817
*Ilanga cf. norfolkensis*	EBISCO/DW2603	Chesterfield, New Caledonia	568–570 m	19°36′S, 158°43′E	MNHN 200718324	HF586129	HF586270	HF585980	HF585814
*Ilanga cf. norfolkensis*	EBISCO/DW2603	Chesterfield, New Caledonia	568–570 m	19°36′S, 158°43′E	MNHN 200718325	HF586130	HF586271	HF585981	HF585815
*Ilanga biradiatula*	MAINBAZA/CP3135	Maputo transect, Mozambique Channel	480–503 m	25°13′S, 35°18′E	MNHN 20098740	HE800723	HE800624	HE800763	HE800674
*Ilanga biradiatula*	MAINBAZA/CP3135	Maputo transect, Mozambique Channel	480–503 m	25°13′S, 35°18′E	MNHN 20098741	HF586133	HF586274	HF585984	HF585818
*Ilanga biradiatula*	MAINBAZA/CP3135	Maputo transect, Mozambique Channel	480–503 m	25°13′S, 35°18′E	MNHN 20098743	HF586134	HF586275	–	HF585819
*Ilanga biradiatula*	MIRIKY/CP3184	Between Nosy-bé and Banc du Leven, Madagascar	492–524 m	12°40′S, 48°12′E	MNHN 20098759	HF586135	HF586276	HF585985	HF585820
*Ilanga* 9	EBISCO/CP2571	N Bellona, New Caledonia	298–309 m	20°25′S, 158°45′E	MNHN 200718314	HF586137	HF586278	HF585987	HF585822
*Ilanga* 9	EBISCO/CP2571	N Bellona, New Caledonia	298–309 m	20°25′S, 158°45′E	MNHN 200718315	HF586138	HF586279	HF585988	HF585823
*Ilanga* 9	EBISCO/DW2618	Lansdowne, New Caledonia	280–304 m	20°06′S, 160°23′E	MNHN 200718326	HF586139	HF586280	HF585989	HF585824
*Ilanga* 9	EBISCO/DW2618	Lansdowne, New Caledonia	280–304 m	20°06′S, 160°23′E	MNHN 200718327	HF586140	HF586281	HF585990	HF585825
*Ilanga* 9	EBISCO/DW2618	Lansdowne, New Caledonia	280–304 m	20°06′S, 160°23′E	MNHN 200718328	HE800725	HE800626	HE800765	HE800676
*Ilanga* 9	EBISCO/CP2571	N Bellona, New Caledonia	298–309 m	20°25′S, 158°45′E	MNHN 200734080	HF586141	HF586282	HF585991	HF585826
*Ilanga* 10	NORFOLK2/DW2135	Norfolk Ridge, Banc Munida, New Caledonia	295–330 m	23°02′S, 168°21′E	MNHN 200718340	–	–	–	HF585827
*Ilanga* 10	TERRASSES/CP3092	SE Terrasses, New Caledonia	360–380 m	22°13′S, 167°12′E	MNHN 20098797	–	–	–	HF585828
*Ilanga* 10	TERRASSES/CP3092	SE Terrasses, New Caledonia	360–380 m	22°13′S, 167°12′E	MNHN 20098798	HF586142	HF586283	HF585992	HF585829
*Ilanga* 10	TERRASSES/CP3092	SE Terrasses, New Caledonia	360–380 m	22°13′S, 167°12′E	MNHN 20098799	HF586143	HF586284	HF585993	HF585830
*Ilanga* 10	TERRASSES/CP3092	SE Terrasses, New Caledonia	360–380 m	22°13′S, 167°12′E	MNHN 20098800	HF586144	HF586285	HF585994	HF585831
*Ilanga* 10	TERRASSES/CP3087	SE Terrasses, New Caledonia	380–400 m	22°11′S, 167°12′E	MNHN 20098805	HF586145	HF586286	HF585995	HF585832
*Ilanga* 10	TERRASSES/CP3087	SE Terrasses, New Caledonia	380–400 m	22°11′S, 167°12′E	MNHN 20098806	HF586146	HF586287	HF585996	HF585833
*Ilanga* 10	TERRASSES/CP3087	SE Terrasses, New Caledonia	380–400 m	22°11′S, 167°12′E	MNHN 20098807	HF586147	HF586288	HF585997	HF585834
*Ilanga* 10	TERRASSES/CP3087	SE Terrasses, New Caledonia	380–400 m	22°11′S, 167°12′E	MNHN 20098808	HF586148	HF586289	HF585998	HF585835
*Ilanga* 10	TERRASSES/DW3079	SE Terrasses, Passe de la Sarcelle, New Caledonia	300–420 m	22°28′S, 167°29′E	MNHN 20098809	HF586149	HF586290	HF585999	HF585836
*Ilanga* 11	MAINBAZA/CP3143	Maputo transect, Mozambique Channel	264–277 m	23°32′S, 35°46′E	MNHN 200915174	HF586106	–	HF585956	HF585890
*Ilanga* 12	PANGLAO2004/T27	Between Panglao I. and Pamilacan I., Philippines	106–137 m	9°33.4′N 123°51.0′E	MNHN 200718221	–	–	–	HF585811
*Ilanga* 12	PANGLAO2004/T27	Between Panglao I. and Pamilacan I., Philippines	106–137 m	9°33.4′N 123°51.0′E	MNHN 200913303	HF586126	HF586267	HF585977	HF585810
*Ilanga gotoi*	PANGLAO2004/T31	Between Panglao I. and Balicasag I., Philippines	100–140 m	9°33.0′N, 123°42.0′E	MNHN 200718349	HF586136	HF586277	HF585986	HF585821
*Ilanga laevissima*	NMDP Africana/St A 18178 D	S of Tsitsikamma, W Cape, South Africa	115 m	34°25′S, 24°00′E	NMSA V3139	–	–	HF586001	–
*Ilanga laevissima*	NMDP Africana/St A 18994 D	Plettenberg Bay, S Cape, South Africa	104 m	34°19.5′S, 23°30′E	NMSA V4397	HF586151	HF586293	HF586003	HF585788
*Ilanga* 15	BIOPAPUA/CP3759	Off Feni Is, Papua New Guinea	287–352 m	04°00′S, 153°36′E	MNHN 200915196	HF586113	HF586252	HF585963	HF585795
*Ilanga* 16	TERRASSES/DW3094	SE Terrasses, New Caledonia	250–300 m	22°04′S, 167°03′E	MNHN 20098801	HF586121	HF586262	HF585972	HF585805
*Ilanga* 17	PANGLAO 2005/CP2393	Bohol Sea, off Balicasag I., Philippines	356–396 m	9°30′N, 123°42′E	MNHN 200735011	–	HF586260	–	–
*Ilanga* 17	PANGLAO2005/CP2332	Bohol Sea, Maribojoc Bay, Philippines	584–596 m	9°38.2′N, 123°43.5′E	MNHN 200718416	HF586115	HF586254	HF585965	HF585797
*Ilanga* 17	PANGLAO2005/CP2331	Bohol Sea, Maribojoc Bay, Philippines	255–268 m	9°39.2′N, 123°47.5′E	MNHN 200718417	HF586116	HF586255	HF585966	HF585798
*Ilanga* 17	PANGLAO2005/CP2340	Bohol Sea, off Balicasag I., Philippines	271–318 m	9°29.4′N, 123°44.4′E	MNHN 200718418	HF586117	HF586256	HF585967	HF585799
*Ilanga* 17	PANGLAO2005/CP2340	Bohol Sea, off Balicasag I., Philippines	271–318 m	9°29.4′N, 123°44.4′E	MNHN 200718419	HF586118	HF586257	HF585968	HF585800
*Ilanga* 17	PANGLAO2005/CP2340	Bohol Sea, off Balicasag I., Philippines	271–318 m	9°29.4′N, 123°44.4′E	MNHN 200718420	HF586119	HF586258	HF585969	HF585801
*Ilanga* 17	PANGLAO2005/CP2344	Bohol Sea, off Pamilacan I., Philippines	128–142 m	9°28.4′N, 123°50.1′E	MNHN 200718421	HF586120	HF586259	HF585970	HF585802
*Ilanga* 17	PANGLAO2005/CP2381	Sill between Bohol and Sulu Seas, Dipolog Bay, Philippines	259–280 m	8°43′N, 123°19′E	MNHN 200735122	–	HF586261	HF585971	HF585804
*Ilanga* 18	NMDP (Africana)/St A 18178 D	S of Tsitsikamma, W Cape, South Africa	115 m	34°25′S, 24°00′E	NMSA V3139	–	–	HF586002	–
*Ilanga* 19	–	Off Shionomisaki, Wakayama Pref., Japan	210 m	33°25′N, 135°41.7′E	No voucher	EU530040	EU530141	–	–
*Ilanga* 20	SALOMON2/CP2287	E Rendova I., Solomon Islands	253–255 m	8°40.8′S, 157°24.6′E	MNHN 200718536	–	–	–	HF585785
*Ilanga* 20	SALOMON2/CP2287	E Rendova I., Solomon Islands	253–255 m	8°40.8′S, 157°24.6′E	MNHN 200913307	–	–	HF585955	HF585883
*Hazuregyra watanabei*	R/V Wakataka-maru, Leg. 3/EF350	Off Kinkazan, Miyagi, Honshu I., Japan	350 m	37º 59′ N, 141º 59′ E	YK1464	HF586105	HF586245	HF585954	HF585784
*“Machaeroplax” delicatus*	R/V Tansei-maru, KT-11-12/T10-2	Off Cape Toi, Miyazaki, Kyushu I., Japan	1063–1082 m	31º 07′ N, 131º 39′ E	YK1484	HF586197	HF586342	HF586048	HF585896
*Minolia nyssonus*	–	Off Kanaya, Chiba, Honshu I., Japan	c. 150–200 m	35º 11′ N, 139º 47′ E	YK1386	–	HF586295	–	–
*Minolia nyssonus*	T/V Seisui-maru, 96-05/D-4	E of Daiozaki, Mie, Honshu I., Japan	263 m	34º 17′ N, 137º 10′ E	YK1355	HF586152	HF586294	HF586004	HF585838
*Minolia punctata*	–	Off Misaki, Kanagawa, Honshu I., Japan	80 m	35º 09′ N, 139º 35′ E	YK1379	HF586155	HF586297	HF586007	HF585841
*Minolia* sp.	–	Off Misaki, Kanagawa, Honshu I., Japan	80 m	35º 09′ N, 139º 35′ E	YK0205	HF586154	AB365226	HF586006	HF585840
*Minolia* sp.	–	Off Zyogashima, Miura, Kanagawa Pref., Japan	–	–	No voucher	HF586153	HF586296	HF586005	HF585839
*Solariella affinis*	R/V “Harry Borthen”	Møre og Romsdal county, Vanylven, Rovdefjorden, NE of Kropperevet, Norway	150–200 m	62°11.45′N, 5°34′E	No voucher	–	–	–	HF585871
*Solariella affinis*	R/V “Harry Borthen”	Møre og Romsdal county, Vanylven, Rovdefjorden, NE of Kropperevet, Norway	150–200 m	62°11.45′N, 5°34′E	NHMUK 20120233	–	HF586321	HF586029	HF585872
*Solariella affinis*	R/V “Harry Borthen”	Møre og Romsdal county, Vanylven, Rovdefjorden, NE of Kropperevet, Norway	150–200 m	62°11.45′N, 5°34′E	NHMUK 20120234	–	–	–	HF585873
*Solariella segersi*	PANGLAO2005/CP2344	Bohol Sea, off Balicasag I., Philippines	128–142 m	9°28.4′N, 123°50.1′E	MNHN 200718422	HF586177	HF586322	HF586030	HF585875
*Solariella segersi*	PANGLAO2005/CP2344	Bohol Sea, off Balicasag I., Philippines	128–142 m	9°28.4′N, 123°50.1′E	No voucher	HF586178	HF586323	HF586031	–
*Solariella chodon*	AURORA/CP2712	Philippines	139–140 m	15°20′N, 121°30′E	MNHN 200718289	HF586179	HF586324	HF586032	–
*Solariella chodon*	PANGLAO2004/T26	Boholi I., Cortes, Philippines	123–135 m	9°43.3′N, 123°48.8′E	MNHN 200718348	HF586180	HF586325	HF586033	–
*Solariella* 3	TERRASSES/DW3109	Munida, Norfolk Ridge, New Caledonia	150–180 m	23°01′S, 168°18′E	MNHN 20098857	HF586182	HF586327	HF586035	HF585877
*Solariella* 3	TERRASSES/DW3109	Munida, Norfolk Ridge, New Caledonia	150–180 m	23°01′S, 168°18′E	MNHN 20098858	HF586183	HF586328	–	HF585878
*Solariella* 3	TERRASSES/DW3109	Munida, Norfolk Ridge, New Caledonia	150–180 m	23°01′S, 168°18′E	MNHN 20098859	HF586184	HF586329	HF586036	HF585879
*Solariella* 3	TERRASSES/DW3109	Munida, Norfolk Ridge, New Caledonia	150–180 m	23°01′S, 168°18′E	MNHN 20098860	HF586185	HF586330	HF586037	HF585880
*Solariella* 4	MAINBAZA/CC3163	Inhambane transect, Mozambique Channel	406–410 m	24°09′S, 35°42′E	MNHN 200915171	HF586192	–	HF586043	HF585894
*Solariella* 4	MAINBAZA/CC3163	Inhambane transect, Mozambique Channel	406–410 m	24°09′S, 35°42′E	MNHN 200915172	–	–	–	HF585888
*Solariella* 4	MAINBAZA/CC3163	Inhambane transect, Mozambique Channel	406–410 m	24°09′S, 35°42′E	MNHN 200915173	–	–	–	HF585889
*Solariella* 4	MAINBAZA/CP3135	Maputo transect, Mozambique Channel	480–503 m	25°13′S, 35°18′E	MNHN 20098745	HF586191	HF586337	HF586042	HF585887
*Solariella* 4	MAINBAZA/CP3135	Maputo transect, Mozambique Channel	480–503 m	25°13′S, 35°18′E	MNHN 20098747	HF586193	–	HF586044	HF585892
*Solariella dedonderorum*	PANGLAO2005/DW2400	Bohol Sea, off Balicasag I., Philippines	111–115 m	9°32.5′N, 123°41.8′E	MNHN 200718427	HF586181	HF586326	HF586034	HF585876
*Solariella* 6	SALOMON2/DW2169	Russel I., W Bay, Solomon Islands	100–200 m	9°01.1′S, 159°5.7′E	MNHN 200718537	–	HF586338	–	HF585891
*Solariella* 7	BERYX/DW18	New Caledonia	250–270 m	24°48′S, 168°09′E	MNHN NR	–	–	–	HF585874
*“Solariella*”*varicosa*	R/V “Asterias”	Finnmark county, Varangerfjorden, SW of Vestre Jakobselv, Norway	10–174 m	70°4′N, 29°12′E	NHMUK 20120235	–	–	–	HF585720
*Spectamen* 1	PANGLAO2004/T39	W Pamilacan I., Cervera Shoal, Philippines	100–138 m	9°30.1′N, 123°50.4′E	MNHN 200718351	HF586186	HF586331	HF586038	HF585881
*Spectamen* 2	T/V Nagasaki-maru/N295, Dredge 1	W of Takarajima I., Tokara Is, Japan	183–184 m	29º 25′ N, 127º 18′ E	YK1381	HF586189	HF586335	HF586040	HF585885
*Spectamen laevior*	PANGLAO2005/CP2344	Bohol Sea, off Balicasag I., Philippines	128–142 m	9°28.4′N, 123°50.1′E	MNHN 200718428	HF586187	HF586332	HF586039	HF585882
*Spectamen laevior*	PANGLAO2005/CP2344	Bohol Sea, off Balicasag I., Philippines	128–142 m	9°28.4′N, 123°50.1′E	MNHN 200913305	–	HF586333	–	HF585726
*Spectamen* 4	CSIRO RV “Southern Surveyor”/SS1005/042	Off Bald I., Western Australia, Australia	973–999 m	35°16.11′S, 118°43.12′	WAM S25789	–	HF586318	–	HF585868
*Spectamen* 4	CSIRO RV “Southern Surveyor”/SS1005/042	Off Bald I., Western Australia, Australia	973–999 m	35°16.11′S, 118°43.12′E	WAM S25789	HF586175	HF586319	HF586027	HF585869
*Spectamen mutabilis*	AURORA/CP2695	Philippines	357–367 m	14°46′N, 123°40′E	MNHN 200718288	HE800727	HE800627	HE800767	HE800678
*Spectamen mutabilis*	AURORA/CP2695	Philippines	357–367 m	14°46′N, 123°40′E	MNHN 200928738	HF586188	HF586334	–	HF585884
*Spectamen mutabilis*	T/V Nagasaki-maru/N319, St. G3	W of Kusagaki Is, Kyushu, Japan	298–299 m	30°39′N, 127°54′E	YK1462	HF586190	HF586336	HF586041	HF585886
*Spectamen philippensis*	–	N Moreton I., Moreton Bay, Queensland, Australia	31 m	26°56.6′S, 153°24.2′E	NHMUK 20110452	EU530042	–	HE800766	–
*Spectamen philippensis*	–	N Moreton I., Moreton Bay, Queensland, Australia	31 m	26°56.6′S, 153°24.2′E	NHMUK 20110452	HF586176	HF586320	HF586028	HF585870
*Spectamen philippensis*	–	N Moreton I., Moreton Bay, Queensland, Australia	31 m	26°56.6′S, 153°24.2′E	NHMUK 20110452	–	–	–	HE800677
*Suavatrochus* sp	T/V Nagasaki-maru, N295/R-2(3)	W of Amami I., Japan	704–730 m	28 36′N, 127 04′E	YK1382	HF586198	HF586343	HF586049	HF585897
*Zetela* 1	MAINBAZA/CP3138	Maputo transect, Mozambique Channel	700–707 m	25°13′S, 35°21′E	MNHN 20098748	HF586195	HF586341	HF586047	HF585895
*Zetela* 1	MAINBAZA/CP3138	Maputo transect, Mozambique Channel	700–707 m	25°13′S, 35°21′E	MNHN 200915167	HF586194	HF586339	HF586045	HF585786
*Zetela* 1	MAINBAZA/CP3138	Maputo transect, Mozambique Channel	700–707 m	25°13′S, 35°21′E	MNHN 200915169	–	HF586340	HF586046	HF585893
*Zetela* 2	ANDEEP III/PS67/074-6-E	Eastern Weddell Sea, Antarctica	1030 m	71°18.35′S, 13°57.71′W	NHMUK 20120236	HF586050	HF586199	HF585898	HF585714
*Zetela* 3	BIOPEARL II/BIO6-AGT-2B	Amundsen Sea, Antarctica	984–1000 m	71°10′S, 109°53′W	NHMUK 20120237	HF586052	HF586201	HF585900	HF585716
*Zetela* 3	LAMPOS ANDEEP/150-1	Burdwood Bank, Antarctica	286–290 m	54°30.22′S, 56°8.2′W	NHMUK 20120238	HF586051	HF586200	HF585899	HF585715
*Zetela* 3	LAMPOS ANDEEP/150-1	Burdwood Bank, Antarctica	286–290 m	54°30.22′S, 56°8.2′W	No voucher	–	HF586202	HF585901	HF585717
*Zetela* 3	LAMPOS ANDEEP/150-1	Burdwood Bank, Antarctica	271–272 m	54°1.36′S, 62°1.33′W	NHMUK 20120239	HF586053	HF586203	HF585902	HF585718
*Zetela* 3	LAMPOS ANDEEP/150-1	Burdwood Bank, Antarctica	286–290 m	54°30.22′S, 56°8.2′W	NHMUK 20120240	HF586054	HF586204	HF585903	HF585719
*Zetela kopua*	RV Tangaroa/2003209	Seamount WNW of Three Kings Is, New Zealand	1145–1185 m	34°2.9′S, 171°8.2′E	NMNZ M160804	HF586196	–	–	–

MNHN, Muséum National d'Histoire Naturelle, Paris; NHMUK, Natural History Museum, London; NMNZ, Museum of New Zealand Te Papa Tongarewa, Wellington; NMSA, KwaZulu-Natal Museum, South Africa; WAM, Western Australian Museum, Perth; YK, personal collection of Yasunori Kano; NR, not registered; GB, GenBank. Note new, corrected locality data for GenBank samples used in Williams et al. (2008). Details for outgroup taxa in Table S1.

### Laboratory methods, sequence editing, and alignment

DNA was extracted from ethanol-preserved foot or mantle tissue (or in a few cases dried specimens) following the protocol described by Williams and Ozawa (2006). The amplification protocols described by Williams et al. (2010) were used to amplify portions of the nuclear 28S rRNA gene (28S: 1496 bp) and three mitochondrial genes: cytochrome oxidase subunit I (COI: 709 bp), 16S rRNA (16S: ∼610 bp) and 12S rRNA (12S: ∼685 bp). Sequence reactions were performed directly on purified PCR products using a BigDye Terminator v1.1 Cycle Sequencing Kit (Applied Biosystems, Foster City, CA) and run on an Applied Biosystems 3730 DNA Analyser automated capillary sequencer. Sequencing and PCR primers are listed in Table S2. Sequences were edited using Sequencher (v. 4.8, Gene Codes Corporation, Ann Arbor, Michigan). A total of 670 sequences were analyzed in this study, of which 631 were new (EMBL accession numbers in Table 1).

Alignment of solariellid COI sequences was performed in MacClade (v 4.08 OSX; Maddison and Maddison 2003). Alignment of COI including outgroups required two insertions, each of a single amino acid for Liotiidae sequences (as previously noted by Kano 2008 and Williams 2012). Ribosomal genes were aligned using MAFFT (v 6.864; Katoh et al. 2002; online: http://mafft.cbrc.jp/alignment/server/). The G-INS-i option was used, which is recommended for sequences with global homology (Katoh et al. 2005), the gap opening penalty was set to 1 and the offset value was set at 0.1, as long gaps were not expected. Scoring matrix for nucleotide sequences were set to “1PAM/κ = 2” for 28S as sequences were very similar, but “20PAM/κ = 2” for mitochondrial ribosomal genes. Poorly aligned sites in rRNA alignments were identified using Gblocks Server (0.91b, Castresana 2000; http://molevol.cmima.csic.es/castresana/Gblocks_server.html) and removed from analyses. Parameters used in Gblocks allowed for smaller final blocks, gap positions within the final blocks and less strict flanking positions.

### Species delimitation

We used the single-threshold, general mixed Yule-coalescent (GMYC) model as implemented by SPLITS (code written by T. Ezard, T. Fujisawa and T. Barraclough in R, v.2.10, http://cran-project) to identify species from sequence variation in mitochondrial genes. We used COI on its own, as COI is commonly used as a “barcoding” gene, but we also used concatenated sequences from all three mitochondrial genes as a previous study on low dispersal species has suggested that combined genes may be more informative than a single gene for species delimitation (Williams et al. 2011). We did not use 28S as the GMYC procedure provides a potential means of detecting species from single-locus sequence data (Monaghan et al. 2009). Instead, we examined the 28S sequences to determine whether any species shared identical genotypes.

Taxon sets differed between the two GMYC analyses. In the combined mitochondrial gene analysis, we used concatenated sequence from all mitochondrial genes including those specimens with two or three mitochondrial sequences. Where preliminary analyses showed sequences formed a tight cluster in independent gene trees, samples from each clade were limited to three specimens in the combined dataset. This dataset included some species that were missing COI data. All individuals with COI sequences were included in the single gene analysis. Eleven specimens were not included in either analysis because of missing data.

Ultrametric trees were produced for GMYC analyses using Bayesian inference as implemented in the program BEAST (v.1.7.1; Drummond and Rambaut 2007) with a relaxed lognormal clock, but without any fossil calibrations and a fixed mean rate of substitutions set to one. We used a constant coalescent prior, which is thought to be more conservative than a Yule prior for delimiting species (Monaghan et al. 2009). Where multiple genes were used, sequence variation was partitioned among genes and gene-specific nucleotide substitution model parameters were used, with each gene allowed to evolve at a different rate. Nucleotide substitution models used in preliminary analyses in BEAST were determined by MrModelTest using the hierarchical likelihood ratio test (v 2.1, J. Nylander, http://www.ebc.uu.se/systzoo/staff/nylander.html). Where multiple models were suggested, the simplest was chosen. The best models for each data set were determined to be HKY + I + G for 16S and 12S and GTR + I + G for COI. Analyses ran for 200,000,000 generations, sampling every 10,000 generations. The final species tree was a maximum clade credibility tree with median node heights based on 18,000 trees. Length of burnin was determined by examination of traces in Tracer (v. 1.5, Drummond and Rambaut 2007; available from http://beast.bio.ed.ac.uk/Tracer).

### Phylogenetic reconstruction

Species trees using individual genes and concatenated sequences from all four genes were produced using Bayesian inference as implemented in MrBayes (v. 3.2.1, Huelsenbeck and Ronquist 2001). Nucleotide substitution models were those used in species delimitation analyses (plus 28S: GTR + G + I). The temperature was lowered to 0.15 to encourage swapping among chains and the propset command was used to increase the proposal probability of the topology parameter (individual gene datasets: ExtTBR(Tau,V); combined dataset: ExtTBR(Tau{all},V{all})) from 5% to 10%. These parameters were chosen based on previous studies of Trochoidea, which showed a large improvement in convergence time and effective sample size (ESS) values using these settings (Williams 2012). Analyses were run for 20,000,000 generations with a sample frequency of 1000. The first ten percent were discarded, so that 18,000 trees were accepted for each run. The datasets were analyzed in two independent runs, and the final tree was computed from the combination of accepted trees from each run (a total of 36,000 trees). Stationarity and convergence between the two runs were determined by examining the potential scale reduction factors (PSRF), standard deviation of split frequencies and by visual examination of.p files in Tracer (v. 1.5; available from http://beast.bio.ed.ac.uk/Tracer).

A chronogram, where branch length corresponds to time, was produced using Species Tree Ancestral Reconstruction (*BEAST). The *BEAST method co-estimates gene trees and a species tree and allows for the incorporation of multiple exemplars of each species and the independent evolution of each gene without fixing a single topology across loci (Heled and Drummond 2010). Two separate *BEAST analyses were undertaken to test how calibrations affect node ages. In one analysis we used an uncorrelated relaxed, lognormal clock with three calibrations based on fossil evidence. In the second, only one was used to date the root (see below for details). Eight independent *BEAST analyses ran between 322,000,000 and 500,000,000 generations with sample frequency of 10,000 for the three calibration analysis. Five independent runs were used in the single calibration analysis. In both cases, the Birth–Death tree prior was used for species-level analyses. Sequence variation was partitioned among genes and gene-specific nucleotide substitution model parameters were used, with each gene allowed to evolve at a different rate. Based on preliminary analyses, we simplified the nucleotide substitution models, using HKY + G + I for all genes, which resulted in improved ESS values. In the *BEAST analysis, we used only solariellid sequences, where each individual had sequence data for 28S and at least two mitochondrial gene sequences. Sequences were assigned to 68 species (not all species were included due to missing data) based on results from species delimitation tests and the number of individuals per species was limited to three to improve computation times. Tree topology was linked for the three mitochondrial genes, as the mitochondrial genome is inherited as a single locus. Default priors were used except for fossil calibrations and ucld.mean priors, which were changed to exponential.

As ages can vary between BEAST and *BEAST analyses (e.g., McCormack et al. 2011), we also ran two analyses with BEAST. As with the *BEAST, one had all three calibrations and one had only the root calibrated. The BEAST analyses ran for 100,000,000 generations with sampling every 10,000 generations. A Birth–Death prior with incomplete sampling was used, with each of the 68 included species represented by a single specimen. Sequences were concatenated and a single tree was produced for the four genes. Substitution models were the same as in *BEAST, but lognormal priors were used for ucld.mean priors.

The final *BEAST species trees and BEAST trees were maximum clade credibility trees with mean node heights based on the remaining trees after burnin of <13% trees in each run. Length of burnin was determined by examination of traces in Tracer.

### Diversification

Plots of the log of the number of lineages against node height (“lineages through time”; LTT) were used to illustrate the rate of diversification using Laser (Rabosky 2006) in R (v. 2.15.0). We used the Constant Rate (CR) test with the gamma-statistic of Pybus and Harvey (2000) to determine whether the LTT plots were consistent with a constant net rate of diversification through time. Allowance was made for incomplete taxon sampling by drawing significance values from simulations using a Monte Carlo constant-rate Test (MCCR; Pybus and Harvey 2000) as implemented in Laser (in R). Sampling was incomplete in this study and it is not known exactly how many species are missing. For instance, species ranges are often quite small, so we assume that sampling in new areas would likely result in the discovery of new species. Moreover, only the IWP was intensively sampled and we are missing species from the Atlantic. To address this issue, we used a range of numbers for the total number of solariellids (100, 200, 300, 600, and 6000) that was likely to encompass the true number of species (we estimate the real number of species in Solariellidae is likely to be closer to 300 species than 100 or 6000).

Three alternative models of lineage accumulation were also used to test the distribution of speciation events over time using models described by Paradis (1997) implemented by the Analyses of Phylogenetics and Evolution package (APE; v. 3.0–5, in R). Model A assumes a constant rate of diversification over time and Model B assumes a gradual change in diversification over time and permits calculation of the parameter β. Values of β < 1 indicate that diversification is increasing, either as a result of increased rates of speciation or decreased rates of extinction, whereas values of β > 1 suggest that diversification is slowing down. Model C assumes that there are two distinct rates of diversification, each with its own rate of speciation before (δ1) and after (δ2) a defined point in time (Tc). We also used the relative cladogenesis test (Purvis et al. 1995) as implemented in R (Geiger package; Harmon et al. 2008) to identify nodes with a significantly increased rate of diversification.

Speciation rates for two genera (*Ilanga* and *Bathymophila*) were calculated using equations from Magallon and Sanderson (2000) as implemented in R (Geiger package). *Bathymophila* was chosen as a clade of interest because its species are distributed in intermediate to deep water. Species in this genus fall into two clades, and all species examined to date in one of these clades are sightless (see Discussion for details). *Ilanga* was chosen as a comparison to *Bathymophila*, because it is a shallow to intermediate depth clade and all species examined to date have pigmented eyes (Herbert 1987; this study). From literature reports, we know that at least 12 species of *Ilanga* (Herbert 1987) and six species of *Bathymophila* were not included in this study (Marshall 1999; Vilvens 2009; S. T. Williams and C. Vilvens, unpubl. data). Even so, the total number of species in either clade is not known, although *Ilanga* overall is likely better sampled than *Bathymophila*.

### Fossil calibrations

We used three fossil records to calibrate the chronograms. In each case, the oldest recognizable member of a clade was used to date the node at the base of the crown group. The lower bound of the age range of a fossil gave the minimum age of the node, while the maximum was estimated as the lower bound of two stages older, which allows both for the uncertainty of the fossil age, and for the incompleteness of the fossil record.

The oldest recorded fossil we could unambiguously compare with Recent Solariellidae was “*Solariella” montsecana* from the Campanian of Torallola, Spain (Kiel and Bandel 2001). This species has axial ribs on the first teleoconch whorl and is quite similar to some specimens in Clade A, but it has axial ribs in the umbilicus and species sampled to date in Clade A do not, so it likely represents a separate genus. We used this fossil record to calibrate the age of the entire ingroup. The clade was constrained to be at least 71 Ma (95% interval: 71.4–89 Ma; mean in real space: 4.18, log stdev: 1, offset: 71).

The second calibration was based on *Solariella* sp. from the latest Oligocene part of the Lincoln Creek Formation in western Washington State, United States of America (Fig. 3, Kiel 2010). This species is similar to *S. affinis* so was used to date the crown of the clade including *S. affinis*, here referred to as *Solariella*. The *Solariella* clade was constrained to be at least 23 Ma (95% interval: 23.2–34 Ma; mean in real space: 2.555, log stdev: 1, offset: 23).

The third calibration was based on *Zetela awamoana* Laws 1939, from the Mount Harris Formation, South Island, New Zealand (Beu and Raine 2009); this fossil is from the Altonian stage of the New Zealand time scale, corresponding to the later half of the Burdigalian (early Miocene) of the international time scale (Hollis et al. 2010). Only one nominal species of *Zetela*, *Z. kopua*, was included in this study. Unfortunately sequence was obtained only from 28S for this specimen, so it was not included in the dated analyses. In the 28S tree, it was sister to an undescribed species from Madagascar (Mainbaza expedition) that based on shell characters we would assign to *Lamellitrochus*, which is a probable synonym of *Zetela* Marshall (1999). We therefore used the calibration to constrain the divergence age between this species (*Zetela* 1) and its sister taxa of two Antarctic species. The two Antarctic species were also tentatively assigned to *Zetela* on the basis of morphological similarity to *Z. kopua* and genetic similarity to *Zetela* 1. The node was constrained to be at least 16.7 Ma (Hollis et al. 2010) (95% interval: 16.7–27.9 Ma; mean in real space: 2.65, log stdev: 1, offset: 16.5).

### Depth data

Depth data were only obtained for species and genera used in this study, as the assignment of species to genera is often uncertain. Data for each species were taken from collecting localities for each specimen used in this study (Table 1), and literature records where more detailed information was available for recognized species (*Ilanga biradiatula, I. discus*, Herbert 1987; “*Solariella” varicosa*, Warén 1993; “*Archiminolia” alabida*, “*A*.” *diadema*, Marshall 1999; *Hazuregyra watanabei*, *Minolia nyssonus*, “*Machaeroplax” delicatus*, Hasegawa 2009; Clade C spp. Vilvens and Williams 2013). Literature records were not used for *Z. kopua* or *Solariella affinis* as there are different “forms” that might represent different species (Warén 1993; Marshall 1999). Neither were they used for *I. laevissima* as the specimens identified in Herbert (1987) represent at least two species (genetic results from this study). Instead, museum collections at the NMSA were re-examined to find new depth data for *I. laevissima s.s* and *Ilanga* 18.

Most information from this study was based on dredge and trawl data and as such there is likely to be some degree of error, as depth data were not based on a point source. This effect was minimized by classifying depth range into one of three groups. Depth ranges were classified as “shallow” if species could be found in water <200 m (on the continental shelf); “deep” if species could be found in water >1050 m (bathyal zone); and “intermediate” if species were collected only in 200–1050 m (on the continental slope). Field observations have shown that “typical” deep-sea fauna (e.g., elasipod holothurians, stalked crinoids, hexactinellid sponges) can occur in the tropics as shallow as 150–180 m (Bouchet et al. 2008) justifying our choice of 200 m as a cut-off for shallow water taxa.

Depth ranges were plotted using Statistica (v.8; StatSoft Inc. 2008). The chronogram was used for ancestral character state reconstructions of depth using likelihood reconstruction methods and the Mk1 model in Mesquite (v. 2.75; Maddison and Maddison 2006, 2011). Only two states (shallow and intermediate + deep) were used in this analysis as only two specimens in the chronogram were collected from the bathyal zone.

## Results

### Species delimitation

A total of 71 evolutionary significant units (ESUs) were recognized as a result of GMYC analyses, with 65 entities being recognized in the GMYC_COI tree and 70 in the GMYC_mt-gene tree (Figures S1 and S2). Taxon sets differed between the two analyses, but where they overlapped, the results were completely congruent, except that two species in the GMYC_COI tree (*Zetela* 3 and *Solariella chodon*) were each recognized as two ESUs in the mt-gene tree. Using the equivalent of a 95% confidence interval, the total number of entities identified ranged from 62 to 70 in the GMYC_COI tree and 69 to 71 in the GMYC_mt-gene tree based on model substitutions at two log-likelihood units from the maximum (C.I.; Monaghan et al. 2009). If the lower C.I. is used to define species in the GMYC_COI tree three pairs of ESUs are combined (*Ilanga* 5 with *Ilanga* 16; *Ilanga* 4 with *Ilanga* 17; and Clade A sp. 5 with Clade A sp. 6). In the mt-gene tree using the lower C.I. limit, *Zetela* 3 and *Solariella chodon* are each recognized as single species, as in the GMYC_COI tree. As previous studies have shown that the number of species is probably overestimated in GMYC analyses of low-dispersal groups (Williams et al. 2011), we conservatively treat *Zetela* 3 and *Solariella chodon* each as a single species.

Eleven specimens were not included in either GMYC analysis because of missing data. These were each recognized as distinct species based on morphological differences and large genetic differences for the genes for which sequence was available (*Archiminolia* 3; *Bathymophila alabida*, *Bathymophila* 12, 14 and 17; Clade B *iridescens*; *Ilanga* 18 and 20; *Solariella* 7; “*Solariella” varicosa*; *Zetela kopua*).

Although slowly evolving, the nuclear 28S rRNA gene is sometimes useful for separating species and we found distinct genotypes for most species. The following pairs or groups shared an identical genotype: *Ilanga* 4, 17 and 19; *Ilanga* 5 and 16; *Ilanga* 11 and *I. laevissima*; *Ilanga* 1, *I. biradiatula* and *I. cf. norfolkensis*; *Ilanga* 3 and 15; Clade C sp. 5 and Clade C sp. 8; *Spectamen* 4 and *S. mutabilis*; and *Minolia nyssonus*, *M. punctata* and *M*. sp.

Overall, we recognized 82 species after GMYC analyses (Table 1) and used these species definitions in the *BEAST analysis. Examination of specimens used in genetic analyses confirmed that most putative species can be distinguished from their sister species morphologically by shell characters. Examination of the chronogram suggests that divergence times between two species pairs are very small (*Ilanga* 4 and 17, 1.22 Myr, HPD: 0.28–2.17; and *Ilanga* 5 and 16; 1.13 Myr, HPD: 0.2–2.03). These same pairs were combined into two single species in the COI tree, when using the lower confidence interval. Further work is needed to test their specific status. A third pair (Clade A sp. 5 and Clade A sp. 6) was not tested in this way as one of the putative species was not included in the dated analyses due to missing data. The status of these two species also needs further testing.

### Phylogenetic analyses

**Figure 1:**
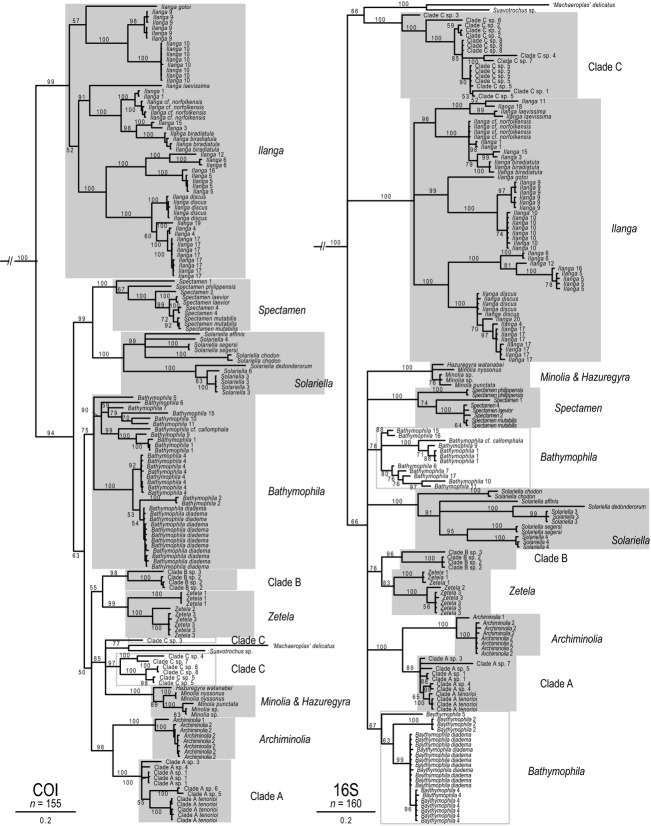
Single gene trees based on Bayesian inference using MrBayes for Solariellidae using mitochondrial genes (16S, cytochrome oxidase subunit I [COI]), with outgroups removed for clarity. Support values are posterior probabilities (PP); branches with PP < 50% were collapsed, PP not shown for intraspecific relationships. See Table 1 for sample details. Monophyletic clades discussed in the text are indicated with a gray shaded box, non-monophyletic groups with a gray outline box.

**Figure 2:**
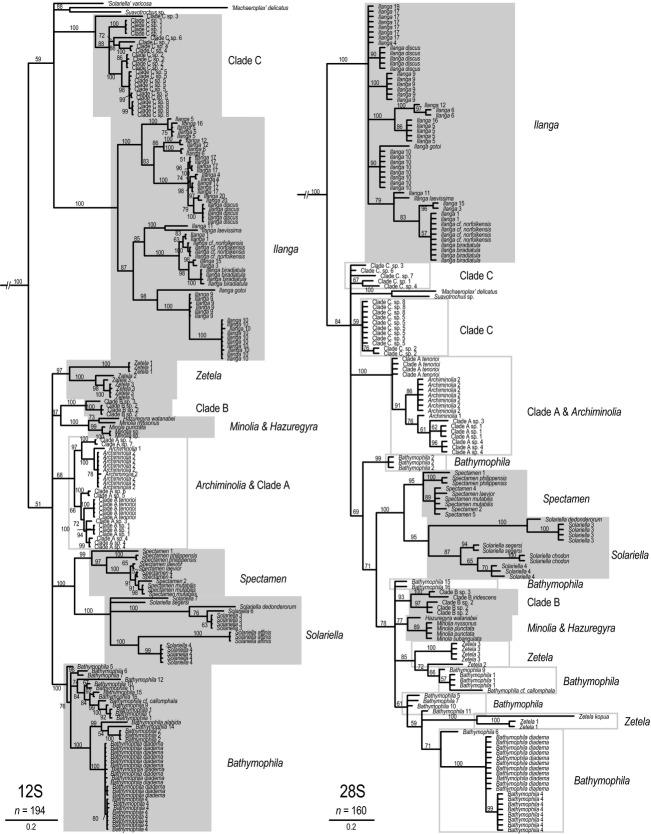
Single gene trees based on Bayesian inference using MrBayes for Solariellidae using the 12S rRNA mitochondrial gene and the 28S rRNA nuclear gene with outgroups removed for clarity. Support values are posterior probabilities (PP); branches with PP < 50% were collapsed, PP not shown for intraspecific relationships. See Table 1 for sample details. Monophyletic clades discussed in the text are indicated with a gray shaded box, non-monophyletic groups with a gray outline box.

**Figure 3:**
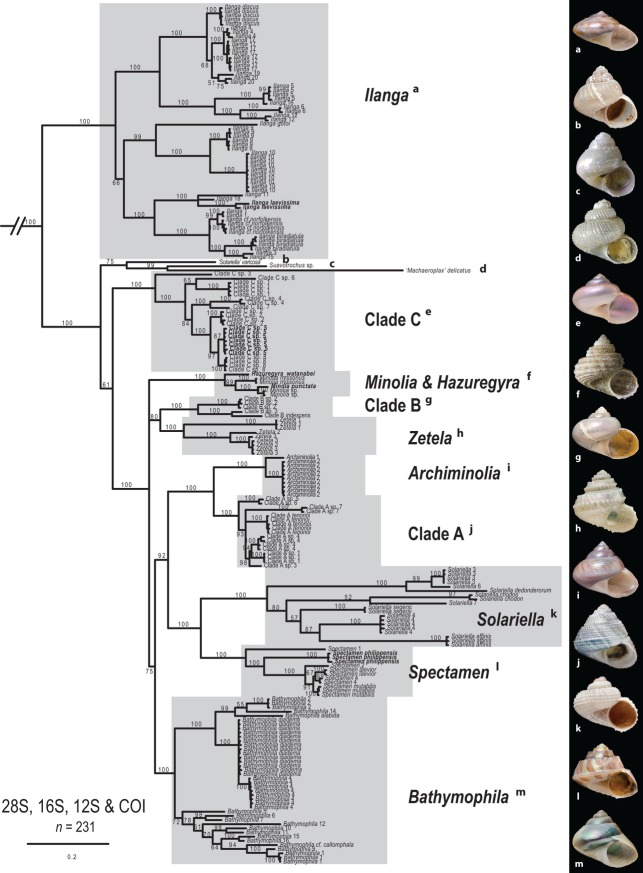
Combined gene tree based on Bayesian inference using MrBayes for Solariellidae using four genes (28S, 16S, 12S and cytochrome oxidase subunit I [COI]), with outgroups removed for clarity (see Figure S3 for outgroup relationships). Support values are posterior probabilities (PP); branches with PP < 50% were collapsed, PP not shown for intraspecific relationships. See Table 1 for sample details. Monophyletic clades discussed in the text are indicated with a gray shaded box. Type species are in bold font. Note that species in Clade C are described by Vilvens and Williams (2013) and assigned to a new genus. Photos are of exemplar species from each clade: (a) *Ilanga biradiatula*; (b) “*Solariella” varicosa*; (c) *Suavotrochus* sp.; (d) “*Machaeroplax” delicatus*; (e) Clade C sp. 8; (f) *Minolia* sp.; (g) Clade B sp. 2; (h) *Zetela* 1; (i) *Archiminolia* 2; (j) Clade A sp. 5; (k) *Solariella affinis*; (l) *Spectamen philippensis;* (m) *Bathymophila* 7.

We obtained well-resolved individual and combined gene trees using MrBayes (Figs. 1–3). In all analyses, average standard deviation of split frequencies approached zero, all parameter average PSRF values were ≤1.001 and minimum ESS values in combined runs exceeded 200 for all parameters. Visual examination of traces showed that all parameters converged between independent runs for each dataset.

**Figure 4:**
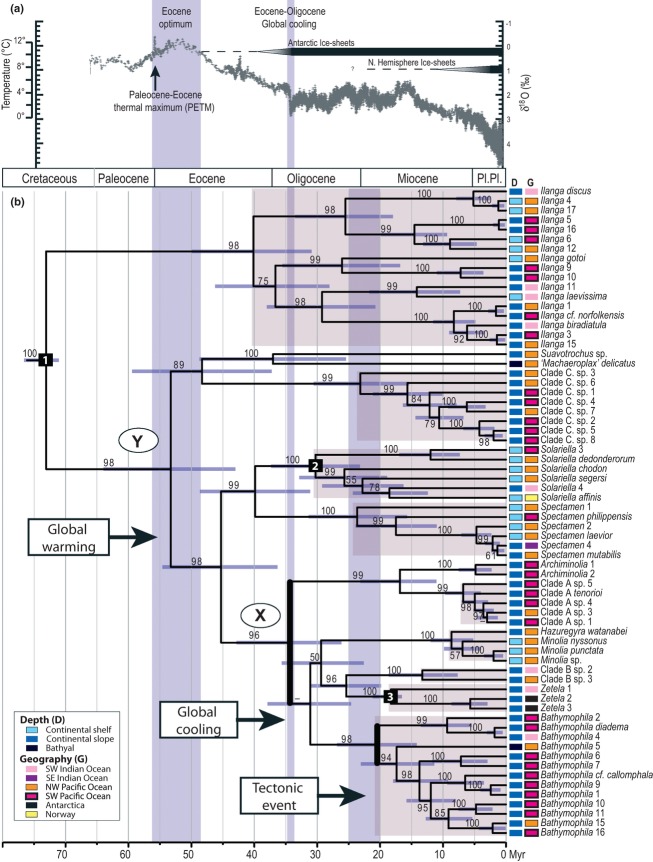
(a) Evolution of global climate over the last 65 Myr. The graph shows a stacked deep-sea benthic foraminiferal oxygen-isotope curve. The δ^18^O temperature scale, on the right axis, applies only to the time preceding the onset of large-scale glaciation on Antarctica (about 35 million years ago). Modified from Figure 2 in Zachos et al. 2008. (b) Chronogram for Solariellidae, with branch lengths proportional to time (scale below in millions of years) based on the three-calibration *BEAST tree. Support values are posterior probabilities (PP, above branches); only values ≥50% are shown. Horizontal, light purple bars on nodes correspond to 95% highest posterior density (HPD) interval for node heights (ages). The 95% HPD is the shortest interval that contains 95% of the sampled values. Clades marked X and Y are discussed in the text. Wide vertical purple bars highlight time periods of interest. Clades with substantially increased rates of diversification are indicated with thickened, vertical black lines. Geographic and depth distributions of species are indicated by a colored box next to the species name (see Key for details). Nodes used to calibrate chronogram are marked with a black square: 1) ingroup calibration; 2) *Solariella* calibration; and 3) *Zetela* calibration.

Ten clades corresponding to genera were recognized in this study. Only three species were not assigned to clades (*Suavotrochus* sp., “*Machaeroplax” delicatus* and “*Solariella” varicosa*). Three genera (*Ilanga, Minolia*, *Spectamen*) and one generic-level clade (Clade B) were recovered as monophyletic in all trees (Figs. 4). Clades A and C were monophyletic in at least two gene trees and the combined tree (Figs. 4). *Archiminolia, Bathymophila* and *Solariella* were not monophyletic as traditionally defined in any tree, but as re-defined in this study *Solariella* was monophyletic in all trees, *Bathymophila* in two gene trees and the combined gene tree and *Archiminolia* in all trees except 28S. Three species that we tentatively assigned to *Zetela* were monophyletic in the combined gene tree, but only *Z. kopua* and *Zetela* 1 formed a clade in the 28S tree (only sequence for 28S was available for *Zetela kopua*). The monotypic *Hazuregyra* was sister to *Minolia* in all analyses.

Outgroup taxa formed well-supported clusters in individual and combined gene trees consistent with families and clades identified in previous studies (Williams 2012) (Figure S3).

### Chronogram

Acceptable ESS values for the *BEAST analysis with three calibrations were obtained by combining 353,662 trees sampled from eight independent runs (ESS >150 for all parameters) and with one calibration by combining 215,331 trees sampled from five independent runs (ESS >200). All ESS values were greater than 200 for both BEAST runs.

The *BEAST tree with three fossil calibrations is shown in Figure 4. Other chronograms are not shown, as the four trees were almost identical in topology, with no well-supported branches (PP > 90%) in conflict. Ages were similar, but consistently younger in analyses with three calibrations rather than with one, both for BEAST and *BEAST (see Table 2 for summary of ages). Moreover, divergence time estimates in *BEAST analyses were generally older than BEAST estimates except for younger clades (particularly nodes <5 Myr). Support values were similar, but slightly lower in *BEAST analyses. Ages used in the Discussion are based on the *BEAST analysis using three calibrations. Relationships among some clades differed slightly between the MrBayes and *BEAST trees, but most of these differences were not well supported.

**Table 2:** Estimated crown ages (and 95% highest posterior density interval) in millions of years for solariellid clades calculated in separate *BEAST or BEAST analyses

Genus/Clade	*BEAST – 3 calibrations	*BEAST – 1 calibration	BEAST – 3 calibrations	BEAST – 1 calibration
Solariellidae	*73.08 Myr (71.09–76.6)	*73.77 Myr (71.07–78.91)	*72.61 Myr (71.05–75.01)	*72.83 Myr (71.75–75.73)
*Archiminolia*	4.82 Myr (2.28–7.49)	5.38 Myr (2.7–8.37)	4.29 Myr (2.44–6.47)	4.66 Myr (2.62–7.12)
*Bathymophila*	20.46 Myr (14.19–26.86)	22.94 Myr (16.11–29.54)	18.91 Myr (14.16–24.28)	20.5 Myr (14.37–27.13)
Clade C	23.16 Myr (15.43–30.55)	25.53 Myr (17.92–34.06)	20.02 Myr (14.01–26.5)	21.5 Myr (14.36–29.27)
*Ilanga*	40.14 Myr (30.9–49.89)	45.53 Myr (35.25–51.84)	34.3 Myr (26.54–41.82)	36.59 Myr (27.71–46.52)
*Minolia*	6.89 Myr (3.97–9.83)	7.49 Myr (4.25–10.79)	6.12 Myr (3.93–8.51)	6.57 Myr (4.16–9.38)
Clade A	6.79 Myr (4–9.68)	7.72 Myr (4.6–11.01)	6.39 Myr (4.18–8.8)	6.95 Myr (4.22–9.74)
Clade B	13.28 Myr (7.67–18.63)	14.58 Myr (8.38–20.84)	11.67 Myr (7.27–16.21)	12.57 Myr (7.39–17.94)
*Solariella*	*30.28 Myr (23.22–37.32)	36.3 Myr (27.06–45.48)	*26.8 Myr (23.12–41.7)	31.06 Myr (22.99–38.88)
*Spectamen*	23.67 Myr (15.73–31.37)	26.9 Myr (17.87–35.85)	20.19 Myr (14.26–26.27)	22.15 Myr (14.71–29.48)
*Zetela*	*18.31 Myr (16.55–21.17)	20.3 Myr (13.71–26.91)	*17.89 Myr (16.58–20.2)	18.93 Myr (12.76–25.49)
Antarctic Clade	5.7 Myr (2.94–8.64)	6.34 Myr (3.24–9.72)	6.69 Myr (4.06–9.66)	7.09 Myr (3.98–10.47)
Clade X	34.33 Myr (26.15–42.84)	37.55 Myr (28.46–47.42)	31.26 Myr (24.5–38.85)	33.68 Myr (24.4–44.17)
Clade Y	53.27 Myr (43.03 –63.99)	58.26 Myr (47.33–68.78)	47.87 Myr (39.02 –57)	52.72 Myr (41.65–63.21)
# unique clades	25,892	22,574	142	141
Highest log clade credibility	−5.98	−5.60	−5.37	−5.00

Nodes used in calibrations marked with an asterisk.

### Diversification

The LTT plot for the solariellid phylogeny was a straightline (not shown), which is the expectation under a constant birth–death model, where the slope equals speciation rate minus extinction rate (Harvey et al., 1994; Pybus and Harvey 2000). The MCCR test confirmed that the LTT plot did not differ significantly from a constant net rate of diversification over time (species sampled = 68; experimental γ = 0.206; number of replicates = 500) in a number of tests with an estimated total number of species ranging between 100 and 6000, thus showing that our result is robust even with the likelihood of missing taxa (range γ_0.05_ = −2.42 to −9.02; *P* range = 0.87 to 1).The survivorship analysis also suggested there was no significant difference between Model A (constant diversification) and Model B (gradual decrease in diversification over time; β = 1.09) (*P* = 0.35) or Model A and C (*P* = 0.58).

However, although the overall rate was constant, the relative cladogenesis test shows that one major clade, designated Clade X in the chronogram (Fig. 4) demonstrated a substantial, although not statistically significant increase in cladogenesis (*P* = 0.08). The most speciose subclade within this clade corresponds to the genus *Bathymophila*. Speciation rates were higher in *Bathymophila* than in the shallower-water genus *Ilanga* over a range of different estimates of total taxa assuming high levels of extinction and almost double when extinction was zero (Table 3).

**Table 3:** Net diversification rate for two solariellid clades based on equations in Magallon and Sanderson (2000), with no extinction (ε = 0) or high extinction (ε = 0.9). N = estimated total number of species in clade, missing = number and percentage not included in this study

	Age (Myr)	ε = 0	ε = 0.9	N (missing)
*Bathymophila*	20.46	0.121	0.056	24 (7, 29%)
		0.138	0.069	34 (17, 50%)
		0.158	0.085	51 (34, 67%)
*Ilanga*	40.14	0.076	0.039	42 (12, 30%)
		0.075	0.038	50 (20, 50%)
		0.085	0.047	60 (40, 67%)

### Depth data

**Figure 5:**
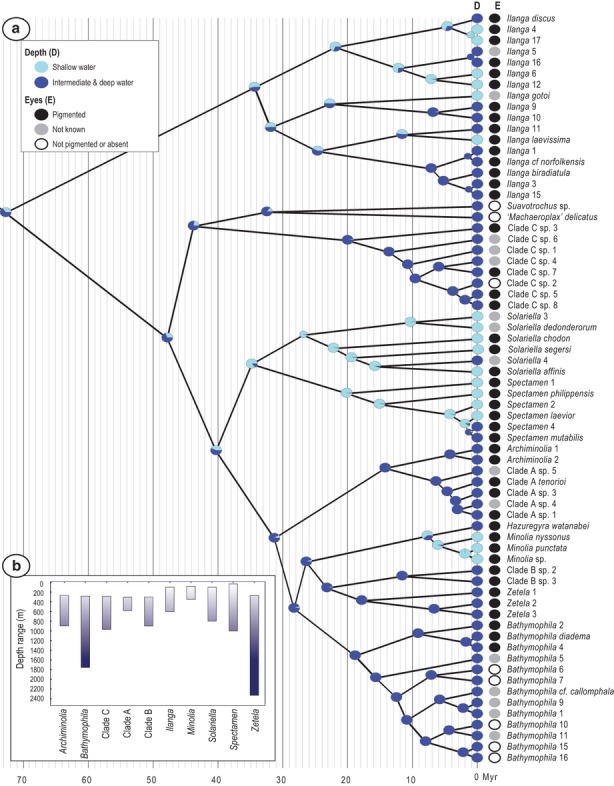
(a) Ancestral state reconstruction of depth distribution in Solariellidae. Ancestral states calculated using the Mk1 model in Mesquite. Pie charts show proportion of likelihood supporting either deep or shallow water habitat. Tree topology is based on the three-calibration *BEAST tree. The presence or absence of eyes is represented graphically next to taxon name. Note that only deep-water taxa are sightless. Light penetration in the ocean varies with latitude and distance from shore. The euphotic zone, where there is sufficient light for photosynthesis to occur, varies in depth, but may extend to around 200 m in the open ocean. (b) Depth ranges for genera and clades discussed in this study, including only species used in this study.

Species were most common on the continental slope (200–1000 m), although the scarcity of both deeper-water species (>1050 m) and shallow species between 50 and 200 m may reflect sampling effort to some extent (Table 1). Combined sampling effort for all stations for the MNHN expeditions listed in this study was greatest in the 200–1050 m range with approximately 78% of stations occurring entirely within these limits (including stations where solariellids were not found). Approximately 17% of stations in these expeditions were all or partly in the range 50–200 m and 5% of stations were partly or entirely in depths >1050 m. Sampling in MNHN expeditions was intense in intertidal and subtidal waters, but in this study, only one solariellid species was found at less than 100 m at a tropical locality (*Spectamen philippensis*), suggesting that solariellids are rare in <50 m in warm, tropical waters, moving into very shallow water only in cooler water (e.g., Japan, Norway, South Africa). Even so, several genera were commonly collected from water defined as shallow for the purposes of this study (<200 m; *Ilanga, Spectamen*, *Solariella* and *Minolia*) (Table 1, Fig. 5). Only three species included in this study were collected at sites >1050 m (*Bathymophila* 5, “*Machaeroplax” delicatus* and *Zetela kopua*) (Table 1, Fig. 5), and few solariellids have been collected alive deeper than 2500 m.

## Discussion

### Systematics

The family Solariellidae is in need of taxonomic revision, with species identification, assignment of species to genera and relationships among genera often uncertain despite several regional monographs that have advanced our understanding of the group (Quinn 1979, 1991; Herbert 1987; Marshall 1999; Vilvens 2009). For instance, of the total 82 species recognized in this study, probably more than two-thirds are either undescribed or have only been described in the last few years. The large number of new species in combination with their patchy distributions suggests that solariellids are extremely diverse and new species are likely to be found as sampling continues. It is important that the systematics of the group is resolved, and this will be addressed separately.

In this study, we note only that ten clades of generic rank were recognized. Contrary to expectation, shell characters could be used to distinguish between most of these clades. This is particularly useful, as many species have been described entirely on the basis of shell characters. Five clades correspond to known genera *Ilanga*, *Spectamen*, *Minolia*, *Zetela* and Clade C (currently being described by Vilvens and Williams 2013). Type species were included for all these genera except *Zetela*. A further two clades of possibly generic rank were identified (Clades A and B).

Clades were also identified that include species assigned to *Solariella*, *Bathymophila* and *Archiminolia*, although no type species were included. If the clades found in this study represent these genera, several species need generic reassignment. Regrettably, the type species of the nominotypical genus *Solariella, S. maculata,* cannot be included as it is a fossil species from the Pliocene. Several North Sea species, especially *S. amabilis* and *S. affinis,* are so similar to *S. maculata*, that they may confidently be considered to represent the genus and further members may yet be found off the coast of West Africa (Herbert 1987; Warén 1993; Marshall 1999). Our study did not include any West African taxa, but did include *S. affinis*. We tentatively assume that the clade including this species corresponds to *Solariella* sensu stricto.

The monotypic *Hazuregyra* was found to be sister and genetically similar to *Minolia*, and should perhaps be considered a synonym thereof. Three species *Suavotrochus* sp., “*Machaeroplax” delicatus* and “*Solariella” varicosa* do not cluster with known genera, but form a poorly supported clade together, sister to Clade C (Fig. 4). Their generic status needs further assessment, preferably including species from the Atlantic. In particular, *Microgaza* (not represented in our dataset) based on a species from the Gulf of Mexico, is conchologically similar to *Ilanga* and the name has been used for Indo-Pacific species, but it differs in radular morphology (Herbert 1987) and its affinities with *Ilanga* remain to be established.

### Origin of deep-sea solariellids

It is not possible to give an accurate estimate of the proportion of total species sampled in this study (we show that there are likely many undescribed species), but species within a genus often share similar biogeographic and depth ranges and we include representatives of all but two currently accepted solariellid genera: the IWP genus *Minolops* (which may be synonymous with *Spectamen*; Marshall 1999) and one predominantly shallow-water Atlantic genus, *Microgaza*. Therefore, although any interpretation of the solariellid phylogeny we present here must be speculative, we can be fairly confident of most patterns observed in this study.

The oldest confirmed fossil solariellids are from shallow tropical settings of Late Cretaceous age (Hickman and McLean 1990; Kiel and Bandel 2001; Kiel et al. 2002). The first records from continental slope palaeodepths are of late Eocene and Oligocene age and were found in cool-temperate regions of both hemispheres (Maxwell 1992; Kiel 2010). The molecular data are not inconsistent with the fossil data, although there is no strong support for either shallow or deep-sea origin. In our tree, the genus *Ilanga* is sister to all other taxa sampled. Although this genus is sister to all other solariellids, ancestral state reconstruction suggests that a deep sea habitat is slightly more plausible for the common ancestor of solariellids (Fig. 5); however, our sampling of shallow water taxa is limited. We have included only 20 of the 32 *Ilanga* species recognized to date (other species listed by Herbert 1987). Of the total number of species, more than half (18) can be found in water <200 m and ten (not included in this study) have only been collected live in <100 m, suggesting that *Ilanga* is a tropical and temperate clade found predominantly in shallow or upper slope waters (50–300 m). The addition of these shallow-water species may change the result, as might the addition of the missing shallow-water genus *Microgaza* or any extinct genera. For instance, the oldest fossil identified is the shallow-water species “*Solariella” montsecana* from the Campanian of Torallola, Spain (Kiel and Bandel 2001). This species, although similar to species in Clade A, probably represents an extinct genus. Shallow, tropical origins of the group as suggested by the fossil data are consistent with patterns showing the tropics and areas with carbonate substrates have acted as cradles of diversity (Jablonski et al. 2006; Alfaro et al. 2007; Kiessling et al. 2010).

Conversely, it has also been suggested that solariellids from Antarctica might be more primitive than previously thought and an Antarctic origin was postulated for the family suggesting the few extant species from Antarctica represent relictual ancestors of lineages that acted as a source of diversity for deep-water communities elsewhere (Linse 2002). This study includes two out of eight recognized Antarctic and sub-Antarctic species, and these form a derived clade within the solariellid tree. The two Antarctic species diverged from their Indian Ocean sister species about 18 Mya (16.55–21.17) during a period of warmer climate (Zachos et al. 2001). This is consistent with the hypothesis that Antarctica acts as a sink for lineages immigrating during warmer periods (Clarke and Crame, 1992; Barnes et al. 2006; Göbbeler and Klussmann-Kolb 2009). The other six Antarctic species not included in this study have been assigned to *Solariella* (Linse 2002). If this assignment is correct, then an Antarctic origin is still unlikely for the family (although possible for the genus).

It has been suggested that deep-sea molluscs have arisen from multiple origins, but at the family and genus levels, the first members of the abyssal fauna to invade the deep sea probably did so in the relatively recent geologic past (Clarke 1962). This suggestion fits with our chronogram, which shows that invasions to the bathyal zone occurred only rarely and since the beginning of the Oligocene (given limited sampling). Invasions into intermediate depth water on the continental slope appear to have occurred more frequently.

The sister clade to *Ilanga* diverged around 53 Mya (HPD: 43.03–63.99; Clade Y, Fig. 4), with lineages in both shallow and deep-water (Figs. 5). Since then, there have been unambiguous invasions from shallow into deeper water (in *Solariella* and *Spectamen*) (Figs. 5). Other invasions are more difficult to interpret. For instance, most likely there was a single transition from deep to shallow water in *Minolia* about 8 or 9 Mya, but there may instead have been two transitions including one from deep to shallow water in the ancestral lineage (perhaps as much as 26–29 Mya) followed by a recent reversal to deep water again in *Hazuregyra*.

Pressure from predators or competitors is unlikely to have played an important role in the invasion of some lineages into deeper water, as specimens with repairs to their shells are frequent, suggesting that mechanical damage, possibly as a result of predation is also common in the deep sea. Equally, bathyal anoxic events probably played a limited role in preventing lineages from diversifying in deep-water in this group, as the radiation of extant taxa is Cenozoic and postdates the most widespread and frequent of these events (Jacobs and Lindberg 1998). Nearly all solariellid specimens from Antarctica collected in this study had highly corroded shells, although this was not evident for species collected at other sites. Arctic species can also show signs of corrosion (A. Waren, pers. obs.). The relevance of these observations, particularly in light of concerns about modern-day ocean acidification, cannot be determined without further work; however, one explanation may be related to the fact that carbon dioxide concentration increases at the poles as a result of decay of organic matter (e.g., Anderson et al. 2010) and over winter as there is virtually no photosynthesis.

### The effect of Cenozoic global climate change on diversification

Climate change is known to be an important factor driving evolution (e.g., Lipps and Mitchell 1976; Berger 2007; Jaramillo et al. 2010). For instance, Vrijenhoek (2013) showed that the crown ages of dominant vent and seep taxa are younger than the PETM, and suggests that they may have radiated after the extinction of earlier lineages. Conversely, the crown age of Solariellidae predates the PETM and although the major solariellid clade sister to *Ilanga* diversified approximately 53 Mya (HPD: 43.03–63.99 Myr; Clade Y, Fig. 4), soon after the PETM (∼55.5 Mya), there is no other molecular evidence in this group for dramatic evolutionary response to this abrupt climate change. This may be because solariellids are most common on the continental slope in depths shallower than 2000 m, and as such were not probably affected by changes to the CCD or deep-sea anoxic events. It is, however, impossible to rule out that some bathyal lineages may have migrated permanently into shallower (continental slope) water or have gone extinct; testing these hypotheses would require detailed fossil evidence.

Another extremely abrupt transition in climate occurred 33.5 to 34 Mya spanning the Eocene–Oligocene boundary, when the Earth abruptly cooled and permanent continental-scale ice sheets first formed in Antarctica (Miller et al. 2009). According to our estimates, solariellid Clade X diversified within this period, approximately 34 Mya (HPD: 26.15–42.84), showing a substantial, although not statistically significant, increase in the rate of cladogenesis. Genera in Clade X are found predominantly in intermediate, slope water or bathyal depths, whereas its sister clade (*Solariella* + *Spectamen*) includes species that can be found in shallow shelf water. A greater diversification of slope rather than shelf species may have been due to a number of factors including an increase in nutrients on the continental slope, especially if food was a limiting factor for slope but not shelf habitats. Nannofossil evidence suggests that ocean productivity increased at intermediate depths (300–500 m) at the EOT in Tanzania, and in the equatorial Pacific and the Southern Ocean (Dunkley Jones et al. 2008; Lyle et al. 2008).

Increased productivity may have arisen as a result of erosion and release of nutrients from organic-rich, shallow, shelf deposits exposed during sea-level falls coincident with large-scale glaciation in Antarctica (Dunkley Jones et al. 2008). Increased ocean circulation at the EOT may also have enhanced production of Subantarctic Mode Water, which transports nutrients from Antarctica to the tropical Indian Ocean (Kiefer et al. 2006) and other tropical/subtropical regions (Dunkley Jones et al. 2008).

In further support of the idea that food may have been a limiting factor, a study on echinoids also showed that while generalist omnivores migrated into deep-water in low numbers over the last 200 Myr, specialist detritivores invaded the deep sea in large numbers between 55 and 75 Mya, probably as a result of increased organic carbon (Smith and Stockley 2005). Solariellids are also specialist deposit feeders; they use highly modified lips to sweep surface detritus into the mouth, and they have greatly shortened radula consistent with little mechanical wear and modified, bifid propodium and mesopodium to facilitate burrowing in soft sediment. Our estimates that the Recent Solariellidae radiated over the last 73 Myr are consistent with the pattern observed in echinoids.

Thus, food availability may have been a factor limiting exploitation of deep-sea habitats for some groups. Other factors, such as increased deep-basin ventilation, a decrease in deep-ocean acidity and a deepening of the CCD by more than 1000 m, which doubled the area of sea-floor subject to calcium carbonate deposition (Rea and Lyle 2005), may have opened up new ecological niches for some groups, allowing invasion of continental shelf and slope lineages into bathyal regions.

### Other factors driving diversification in the deep sea

The increase in diversification in Clade X is due largely to its most speciose subclade, the genus *Bathymophila*. Several factors may have affected diversification in this genus. The *Bathymophila* clade diversified 20.46 Myr (HPD: 14.19–26.86), soon after the collision of the Australia and New Guinea plate with the southeast extremity of the Eurasian plate and the Philippines-Halmahera-New Guinea arc system ∼25 Mya (Hall 1998). This tectonic activity has been invoked as an important driver of speciation 20–25 Mya in shallow-water invertebrates (Kohn 1990; Wilson and Rosen 1998; Williams 2007; Renema et al. 2008; Williams and Duda 2008; Bellwood et al. 2012) and more recently in deep-water organisms (Cabezas et al. 2012) predominantly through the increased availability of new habitats and greater habitat complexity. Another possibility is that terrestrial run-off from the uplift of landmasses and concurrent volcanism provided additional food sources for deep-sea benthic fauna.

A different explanation might be suggested by a study that showed that speciation occurred more rapidly in deep-sea, eyeless clades of ostracods than shallow-water sighted clades (Syme and Oakley 2012). Eyes are unpigmented in several deep-slope and bathyal solariellids (e.g., “*Machaeroplax” delicatus*, *Suavotrochus* sp., Clade C sp. 2*, Bathymophila* 6, 7, 10, 15 and 16). In fact, all species corresponding to Marshall's (1999) concept of *Bathymophila* that have been examined have unpigmented eyes (this study; Marshall 1999), suggesting that it is a common condition for this clade. Like ostracods, diversification also appears to be higher in *Bathymophila* than other shallower, sighted clades. For example, speciation rates are up to double those in *Ilanga*, the most speciose clade sampled in this study with pigmented eyes and found in shallower water (Herbert 1987; Table 3). Key innovations are known to affect rates of diversification (Heard and Hauser 1995) and the loss of a character that no longer offers a selective advantage may also be viewed as an innovation (e.g., Jeffery 2005, 2009). Alternatively, the factor driving diversification in these groups may actually be the deep-water habitat, rather than the loss of eyes per se, as the two are often coupled.

### Species ranges and biogeographic patterns

Solariellids have been shown to have exceptionally patchy distributions, suggesting highly specific ecological requirements (Marshall 1999). No species is known to be endemic to hot vents (Kiel 2006, 2010; Sellanes et al. 2008), although one species has been collected from cold seeps off Chile (Warén et al. 2011). Species used in this study were predominantly collected from soft sediment, were often rare, and more than half the species were found at only one station. In some cases, this probably reflects sampling effort; for example, many deeper-water species (>1050 m) were collected only once and sampling at these depths was more limited. Several species, however, were found at multiple stations within the IWP where sampling effort was concentrated. In every case, these stations with shared taxa were located within a single biogeographic zone (southwest Pacific, northwest Pacific, southwest Indian Ocean or southeast Indian Ocean), although one southeast Indian Ocean species (Clade A sp. 6) was genetically very similar to a species from northwest Pacific (Clade A. sp. 5). No species in this study are shared between southwest and northwest Pacific sites.

The division between southwest Pacific sites (including Eastern Australia, Papua New Guinea, Solomon Islands, Fiji, Vanuatu, Tuamotus, New Zealand) and northwest Pacific sites (including Japan, Taiwan and Philippines) has been observed at both the level of population structure in many highly dispersive, shallow-water species and in the distributions of some deep-sea species (e.g., Macaranas et al. 1992; McMillan and Palumbi 1995; Palumbi 1997; Williams and Benzie 1997; Planes and Fauvelot 2002; Barber et al. 2006; Imron et al. 2007; Magsino and Juinio-Meñez 2008; Lorion et al. 2010). The congruence of pattern is likely the result of the flow of equatorial currents in the Pacific. For high-dispersal, shallow-water species, surface currents act as a porous barrier by redirecting larvae and limiting direct gene flow between southern and northern Pacific sites. However, for deep-water groups with more modest dispersal potential, the eastward-flowing Equatorial Undercurrent, which flows most strongly at the thermocline (100–200 m) (Jewell 1995), is probably more important. The current is likely to have a strong influence on gene flow in solariellids as they have relatively short-lived lecithotrophic larvae (and sometimes brood larvae) (Herbert 1987; Marshall 1999). More highly dispersing species may find the Equatorial Undercurrent a porous barrier. For example, some moderately deep-water species of *Bursa* (a gastropod genus with teleplanic planktotrophic larvae) span the equator, occurring in both the Philippines and New Caledonia or the Philippines and the Solomon Islands (Castelin et al. 2012). A more profound barrier is likely the oxygen minimum zone below the Equatorial Undercurrent (300–400 m; Levitus 1982), which is most pronounced in the Eastern Pacific (Jewell 1995; Levin 2003). These factors combined probably serve as an effective barrier to dispersal of some deep-water taxa across the equator promoting allopatric speciation within biogeographic zones (Wilson 1999; Rogers 2000; McClain and Hardy 2010). The existence of several NW/SW Pacific species pairs supports this idea (*Ilanga* 1/*I. cf*. *norfolkensis* and *Ilanga* 3/15, Clade A sp. 1/3, Clade C sp. 4/7, *Bathymophila* 15/16).

## Supporting Information

**Figure S1.** Results of GMYC test on an ultrametric tree produced using BEAST for the cytochrome oxidase I sequences. (A) Tree with ESUs and species names marked on the right-hand side. Support for nodes are posterior probabilities, shown only for species-level relationships and PP > 50%. Branches in red indicate population structure. (B) Maximum likelihood plot showing peak congruent with threshold limit in B. (C) Lineage through time plot with red line showing the threshold level suggested by GMYC.

**Figure S2.** Results of GMYC test on an ultrametric tree produced using BEAST for concatenated sequences from all three mitochondrial genes. (A) Tree with ESUs and species names marked on the right-hand side. Support for nodes are posterior probabilities, shown only for species-level relationships and PP>50%. Branches in red indicate population structure. (B) Maximum likelihood plot showing peak congruent with threshold limit in B. (C) Lineage through time plot with red line showing the threshold level suggested by GMYC.

**Figure S3.** Combined gene tree based on Bayesian inference using MrBayes for Solariellidae using four genes (28S, 16S, 12S and COI), showing only relationship among outgroups and Solariellidae (solariellid clade is collapsed). Support values are posterior probabilities (PP); branches with PP < 50% were collapsed, PP not shown for intraspecific relationships. See Table S1 for outgroup sampling details. Families and relationships are consistent with those discussed in Williams (2012).

**Table S1.** Outgroup specimens used in study, ordered by family, along with details of sampling localities, registration numbers of voucher specimens, and EMBL accession numbers for sequences. MNHN, Muséum National d'Histoire Naturelle, Paris; NHMUK, Natural History Museum, London; and NSMT, National Museum of Nature and Science, Tokyo, Japan (NSMT). Photos of specimens are in Williams (2012) or available on MorphoBank online at http://www.morphobank.org/index.php/Projects/ProjectOverview/project_id/223.

**Table S2.** Forward (F) and reverse (R) PCR primers (also used in sequencing), and forward (FS) and reverse (RS) internal sequencing primers. Annealing temperatures and concentration of magnesium chloride (MgCl2) used in 50 μL polymerase chain reactions.
