# Supplementary figures and images for: Cenozoic climate change and diversification on the continental shelf and slope: evolution of gastropod diversity in the family Solariellidae (Trochoidea)
Source: Ecol Evol. 2013 Mar 4;3(4):887–917. doi: 10.1002/ece3.513 (PMC3631403; doi:10.1002/ece3.513)

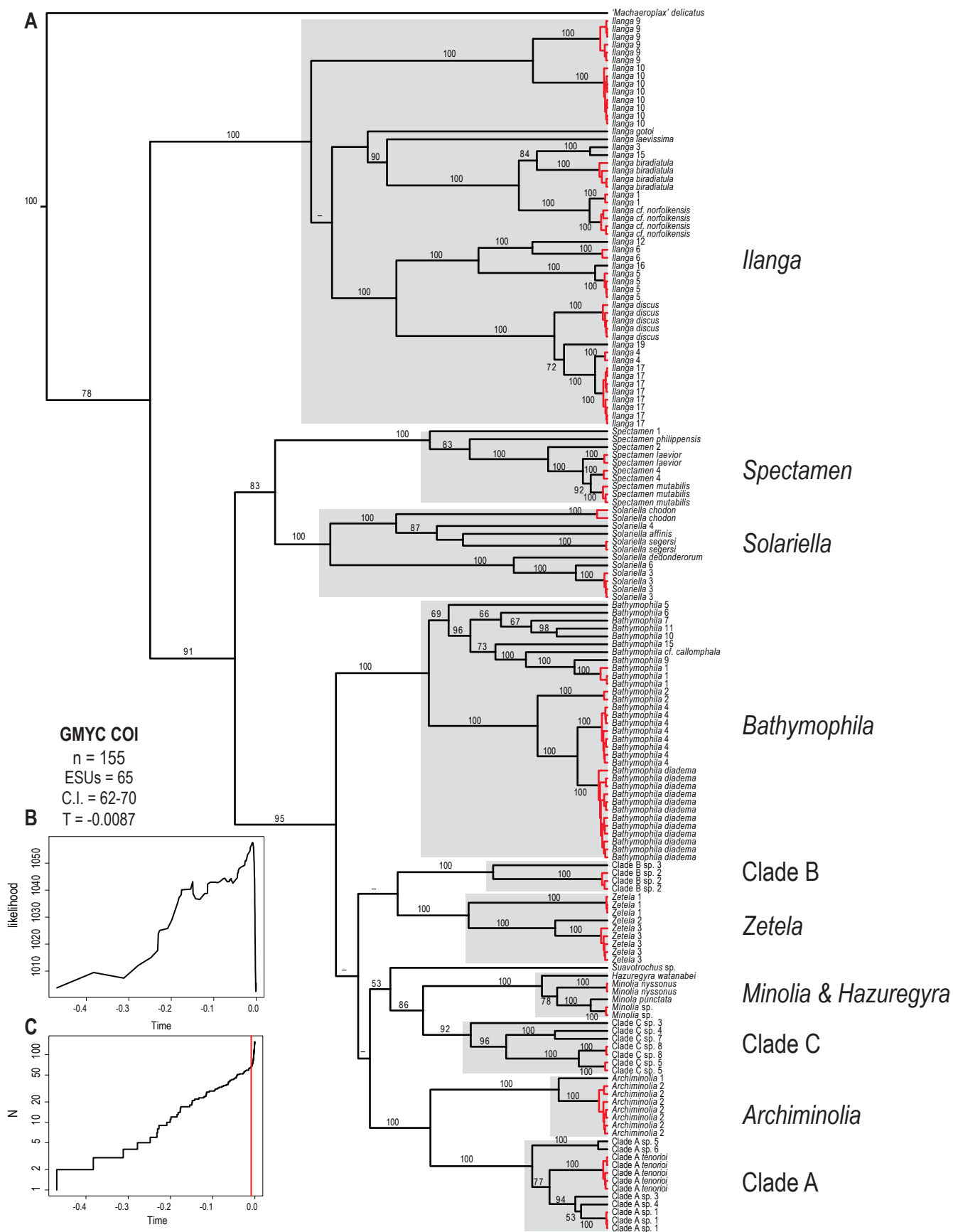

Supplement: Supplementary file 1 [file ece30003-0887-SD1.pdf]

A

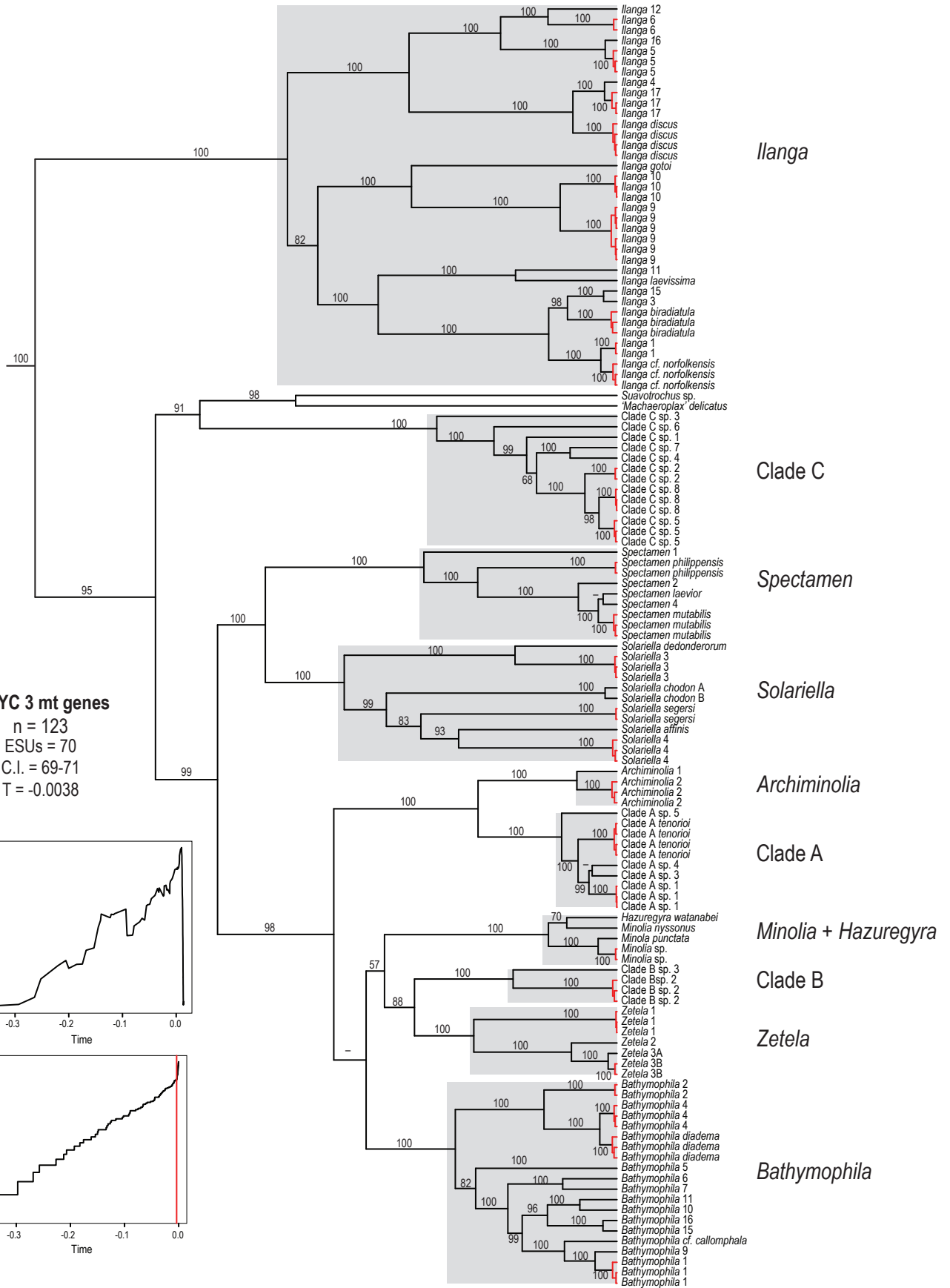

B

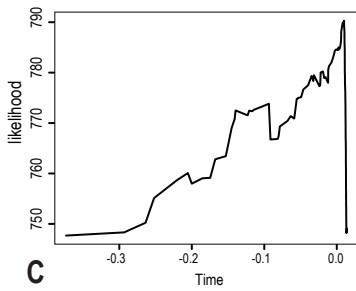

C

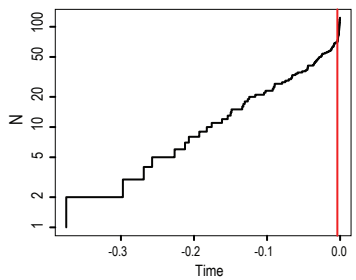

Supplement: Supplementary file 2 [file ece30003-0887-SD2.pdf]

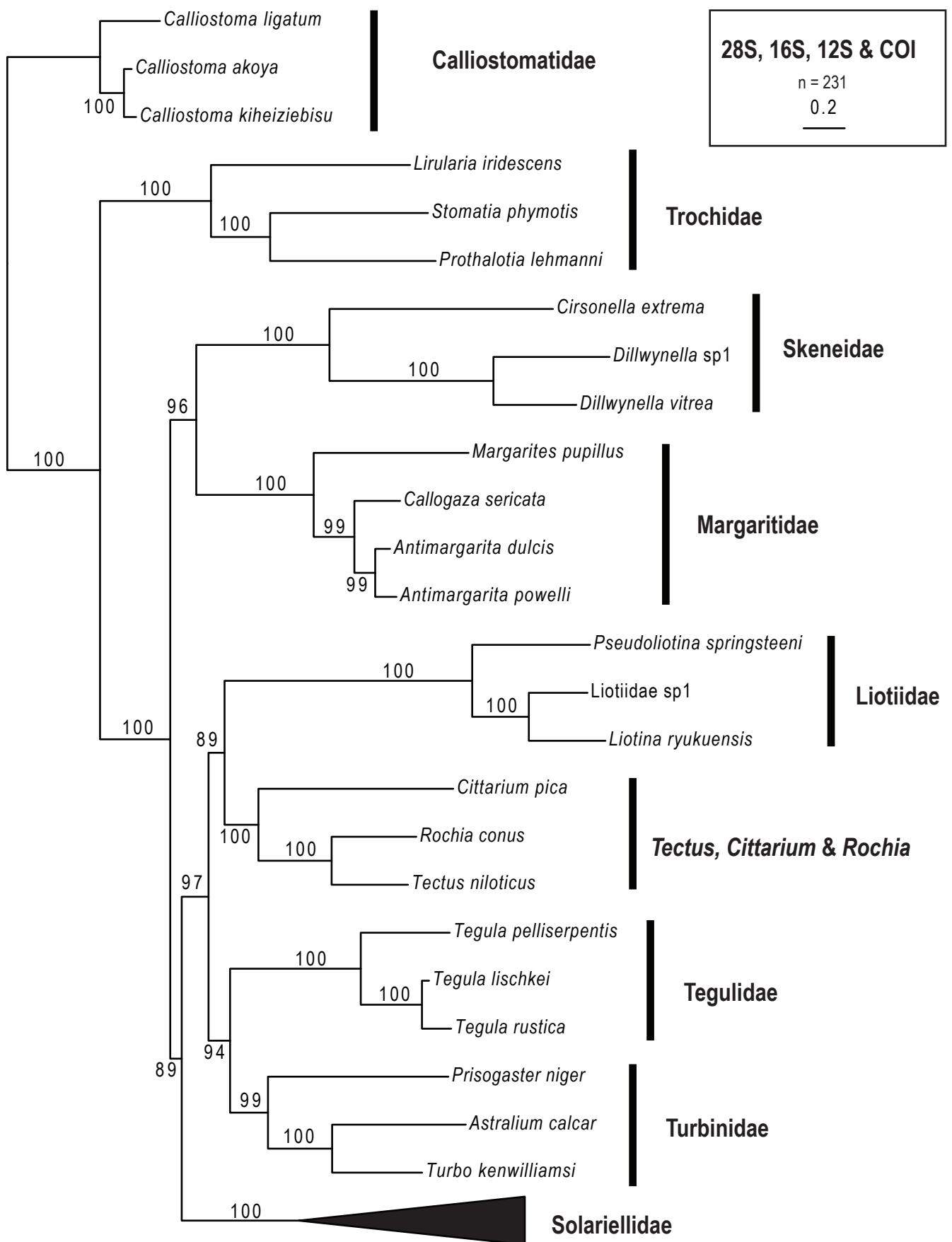

Supplement: Supplementary file 3 [file ece30003-0887-SD3.pdf]
