# Supplementary material for: Cenozoic climate change and diversification on the continental shelf and slope: evolution of gastropod diversity in the family Solariellidae (Trochoidea)
Source: Ecol Evol. 2013 Mar 4;3(4):887–917. doi: 10.1002/ece3.513 (PMC3631403; doi:10.1002/ece3.513)
Supplement: Supplementary file 7 [file ece30003-0887-SD7.doc]

Table S2: Forward (F) and reverse (R) PCR primers (also used in sequencing), and forward (FS) and reverse (RS) internal sequencing primers. Annealing temperatures and concentration of magnesium chloride (MgCl_2_) used in 50 µl polymerase chain reactions.

| Name | Sequence 5′-3′ | Annealing Temperature | MgCl_2_ concentration | Source |
| --- | --- | --- | --- | --- |
|  |  |  |  |  |
| 28S rRNA |  | 52°C | 2.5 mM |  |
| LSU5 (F) | TAG GTC GAC CCG CTG AAY TTA AGC A |  |  | Littlewood et al. 2000 |
| LSU1600R (R) | AGC GCC ATC CAT TTT CAG G |  |  | Williams et al. 2003 |
| 900F (FS) | CCG TCT TGA AAC ACG GAC CAA G |  |  | Lockyer et al. 2003 |
| ECD2S (RS) | CTT GGT CCG TGT TTC AAG ACG G |  |  | Modified from Littlewood et al. 2000 |
| LSU330F (FS) | CAA GTA CCG TGA GGG AAA GTT G |  |  | Littlewood et al. 2000 |
| COI |  | 40-50°C | 3.0 mM |  |
| LCO1490 (F) | ggt caa caa atc ata aag ata ttg g |  |  | Folmer et al. 1994 |
| HCO2198 (R) | TTA ACT TCA GGG TGA CCA AAA AAT CA |  |  | Modified from Folmer et al. 1994 |
| LCOmod (F) | TCT ACT AAT CAT AAG GAY ATY GGN AC |  |  | Kano, 2008 |
| HCOmod (R) | ACT TCT GGG TGT CCR AAR AAY CAR AA |  |  | Kano, 2008 |
| 16S rRNA |  | 50°C | 3.0 mM |  |
| 16Sar-L (F) | CGC CTG TTT ATC AAA AAC AT |  |  | Palumbi et al. 1991; Palumbi 1996 |
| 16Sbr-H (R) | CCG GTC TGA ACT CAG ATC ACG T |  |  | Palumbi et al. 1991; Palumbi 1996 |
| CGLeu^UUR^R (R) | TAT TTA GGG CTT AAA CCT AAT GCA C |  |  | Hayashi, 2005 |
| **12S rRNA** |  | 50-65°C | 2.0 mM |  |
| 12S-I (F) | TGC CAG CAG YCG CGG TTA |  |  | Oliverio & Mariottini, 2001 |
| 12S (-) (R) | AGA GYG RCG GGC GAT GTG T |  |  | Bandyopadhyay *et al.*, 2008 |

REFERENCES

Bandyopadhyay, P.K., Stevenson, B.J., Ownby, J.-P., Cady, M.T., Watkins, M. & Olivera, B.M. 2008. The mitochondrial genome of *Conus textile*, coxI–coxII intergenic sequences and Conoidean evolution. *Molecular Phylogenetics and Evolution,* **46**, 215-223.

Folmer, O., Black, M., Hoeh, W., Lutz, R., Vrijenhoek, R. 1994. DNA primers for amplification of mitochondrial cytochrome c oxidase subunit I from diverse metazoan invertebrates. *Molecular Marine Biology and Biotechnology*, **3**, 294-299.

Hayashi, S. 2005. The molecular phylogeny of the Buccinidae (Caenogastropoda: Neogastropoda) as inferred from the complete mitochondrial 16S rRNA gene sequences of selected representatives. *Molluscan Research,* **25**, 85–98.

Kano, Y. 2008. Vetigastropod phylogeny and a new concept of Seguenzioidea: independent evolution of copulatory organs in the deep-sea habitats. *Zoologica Scripta*, **37**: 1–21.

Littlewood, D.T.J., Curini-Galletti, M., Herniou, E. A. 2000. The interrelationships of *Proseriata* (Platyhelminthes: Seriata) tested with molecules and morphology. *Molecular Phylogenetics and Evolution,* **16**, 449-466.

Lockyer, A.E., Olson, P.D., Ostergaard, P., Rollinson, D., Johnston, D.A., Attwood, S.W., Southgate, V.R., Horak, P., Snyder, S.D., Le, T.H., Agatsuma, T., McManus, D.P., Carmichael, A.C., Naem, S., Littlewood, D.T.J. 2003. The phylogeny of the Schistosomatidae based on three genes with emphasis on the interrelationships of *Schistosoma* (Weinland, 1858). *Parasitology*, **126**, 203–224.

Oliverio, M., Mariottini, P. 2001. A molecular framework for the phylogeny of *Coralliophila* and related muricoids. *Journal of Molluscan Studies*, **67**, 215–224.

Palumbi, S.R., Martin, A., Romano, S., McMillan, W.S., Stice, S. and Grabowski, G. 1991. *The Simple Fool's Guide to PCR*. University of Hawaii Press, Honolulu, HI.

Palumbi, S. R. 1996. Nucleic acids II: the polymerase chain reaction. In: Hillis, D. M., Moritz, C. Mable, B. K. (Eds), *Molecular systematics*. Sinauer Associates, Sunderland, pp. 205-247.

Williams, S.T., Reid, D.G., Littlewood, D.T.J. 2003. A molecular phylogeny of the Littorininae (Gastropoda: Littorinidae): unequal evolutionary rates, morphological parallelism and biogeography of the Southern Ocean. *Molecular Phylogenetics and Evolution,* **28**, 60-86.

Winnepenninckx, B.M.H., Reid, D.G., Backeljau, T. 1998. Performance of 18S rRNA in littorinid phylogeny (Gastropoda: Caenogastropoda). *Journal of Molecular Evolution*, **47**, 586–596.
