# Supplementary material for: Cenozoic climate change and diversification on the continental shelf and slope: evolution of gastropod diversity in the family Solariellidae (Trochoidea)
Source: Ecol Evol. 2013 Mar 4;3(4):887–917. doi: 10.1002/ece3.513 (PMC3631403; doi:10.1002/ece3.513)
Supplement: Supplementary file 8 [file ece30003-0887-SD8.docx]

**Table S1.** Outgroup specimens used in study, ordered by family, along with details of sampling localities, registration numbers of voucher specimens, and EMBL accession numbers for sequences. MNHN: Muséum National d'Histoire Naturelle, Paris; NHMUK: Natural History Museum, London; and NSMT: National Museum of Nature and Science, Tokyo, Japan (NSMT). Photos of specimens are in Williams (2012) or available on MorphoBank online at http://www.morphobank.org/index.php/Projects/ProjectOverview/project_id/223.

| **SPECIES** | **SAMPLING LOCALITY** | **REG. NO.** | **28S** | **COI** | **16S** | **12S** |
| --- | --- | --- | --- | --- | --- | --- |
| **FAMILY CALLIOSTOMATIDAE THIELE, 1924** |  |  |  |  |  |  |
| *Calliostoma kiheiziebisu* Otsuka, 1939 | Off Kesennuma, Miyagi Pref., Japan | – | AB505228 | AB505274 | AB505319 | AB505363 |
| *Calliostoma ligatum* (Gould, 1849) | Friday Harbour, Washington, USA | NHMUK 20080939 | GQ232383 | GQ232357 | GQ232285 | GQ232320 |
| *Calliostoma akoya* (Kuroda in Ikebe, 1942) | Off Hota, Chiba Pref., Japan | NSMT Mo76812 | AB505226 | AB505272 | AB505317 | AB505361 |
|  |  |  |  |  |  |  |
| **FAMILY LIOTIIDAE ADAMS & ADAMS, 1854** |  |  |  |  |  |  |
| *Pseudoliotina springsteeni* McLean, 1988 | Momo Beach, Panglao I., Philippines, 3-14 m | – | HE800698 | HE800601 | HE800745 | HE800645 |
| Liotiidae sp. 1 | Pamilacan I., Philippines, 6-14 m | – | HE800696 | HE800599 | – | HE800643 |
| *Liotina ryukuensis* (Habe, 1991) | Koza, Okinawa Pref., Japan | NHMUK 20050443 | AM048726 | – | AM048897 | HE800644 |
|  |  |  |  |  |  |  |
| **FAMILY MARGARITIDAE THIELE 1924** |  |  |  |  |  |  |
| *Margarites pupillus* (Gould, 1849) | Friday Harbour, Washington, USA | NHMUK 20110448 | EU530045 | HE800613 | HE800752 | HE800659 |
| *Callogaza sericata* (Kira, 1959) morph 1 | Isle of Pines, New Caledonia | MNHN 20098862 | HE800703 | HE800606 | – | HE800650 |
| *Antimargarita dulcis* (Smith, 1907) | E Weddell Sea, Antarctica | NHMUK 20110461 | HE800700 | HE800603 | HE800747 | HE800647 |
| *Antimargarita powelli*  Aldea, Zelaya & Troncoso, 2009 | S Bellingshausen Sea, Antarctica | NHMUK 20110463 | HE800702 | HE800605 | HE800749 | HE800649 |
|  |  |  |  |  |  |  |
| **FAMILY SKENEIDAE CLARK, 1851** |  |  |  |  |  |  |
| *Cirsonella extrema* Thiele, 1912 | South Sandwich Is., Antarctica | NHMUK 20110464 | HE800714 | HE800615 | HE800754 | HE800663 |
| *Dillwynella* sp. 1 | Bohol/Sulu seas sill, Philippines | MNHN 18414 | HE800715 | HE800617 | HE800756 | HE800665 |
| *Dillwynella vitrea* Hasegawa, 1997 | Owase City, Mie Pref., Japan | – | AM048701 | AM049336 | HE800757 | HE800666 |
|  |  |  |  |  |  |  |
| **FAMILY TEGULIDAE KURODA, HABE & OYAMA, 1971** |  |  |  |  |  |  |
| *Tegula pelliserpentis* Wood, 1828 | Guanacaste, Costa Rica | NHMUK 20050730 | EU530056 | EU530153 | HE800777 | HE800684 |
| *Tegula lischkei* Tapparone-Canefri, 1874 | Amiji Is., Oshika City, Miyagi Pref., Japan | NHMUK 20050474 | EU530047 | EU530144 | HE800776 | HE800683 |
| *Tegula rustica* (Gmelin, 1791) | Omaezaki, Shizuoka Pref., Japan | NHMUK 20110455 | EU530050 | EU530147 | HE800778 | – |
|  |  |  |  |  |  |  |
| **FAMILY TROCHIDAE RAFINESQUE, 1815** |  |  |  |  |  |  |
| *Lirularia iridescens* (Schrenck, 1863) | Monbetsu, Monbetsu City, Hokkaido, Japan | NHMUK 20080953 | GQ232397 | EU530125 | GQ232301 | GQ232334 |
| *Stomatia phymotis* Helbling, 1779 | Santo, Vanuatu | MNHN 18155 | GQ232403 | GQ232372 | GQ232306 | GQ232342 |
| *Prothalotia lehmanni* (Menke, 1843) | Dunsborough, Western Australia | NHMUK 20070156 | EU530021 | EU530123 | GQ232302 | GQ232338 |
|  |  |  |  |  |  |  |
| **TROCHOIDEA: FAMILY UNCERTAIN** |  |  |  |  |  |  |
| *Cittarium pica* (Linnaeus, 1758) | Akumal, S of Cancun, Quintana Roo, Mexico | – | EU530049 | EU530146 | HE800768 | HE800679 |
| *Rochia conus* (Gmelin, 1791) | Panglao, Philippines | MNHN 18387 | HE800728 | HE800628 | HE800769 | HE800680 |
| *Rochia niloticus* (Linnaeus, 1767) | Îlot de Tiberama, Poindimié, North Province, New Caledonia | NHMUK 20070142 | EU530054 | EU530151 | HE800772 | HE800682 |
